# Supplementary material for: Copper-catalyzed S-arylation of Furanose-Fused Oxazolidine-2-thiones
Source: Molecules. 2022 Aug 30;27(17):5597. doi: 10.3390/molecules27175597 (PMC9457760; doi:10.3390/molecules27175597)
Supplement: Supplementary file 1 [file molecules-27-05597-s001.zip › molecules-1866030-supplementary.pdf]

Supplementary Materials

Copper-catalyzed *S*-arylation of carbohydrate-fused oxazolidine-2-thiones

Vilija Kederienė [1,*], Jolanta Rousseau [2], Marie Schuler [3], Algirdas Šačkus [1] and Arnaud Tatibouët [3,*]

[1] Department of Organic Chemistry, Kaunas University of Technology, Radvilėnų pl. 19, LT-50254 Kaunas, Lithuania

[2] Univ. Artois, CNRS, Centrale Lille, Univ. Lille, UMR 8181 – UCCS – Unité de Catalyse et Chimie du Solide, F-62300 Lens, France

[3] Université d'Orléans et CNRS, ICOA, UMR 7311, BP 6759, F-45067 Orléans France

***** Corresponding authors

E-mail address: vilija.kederiene@ktu.lt (V. Kederienė); arnaud.tatibouet@univ-orleans.fr (A. Tatibouët)

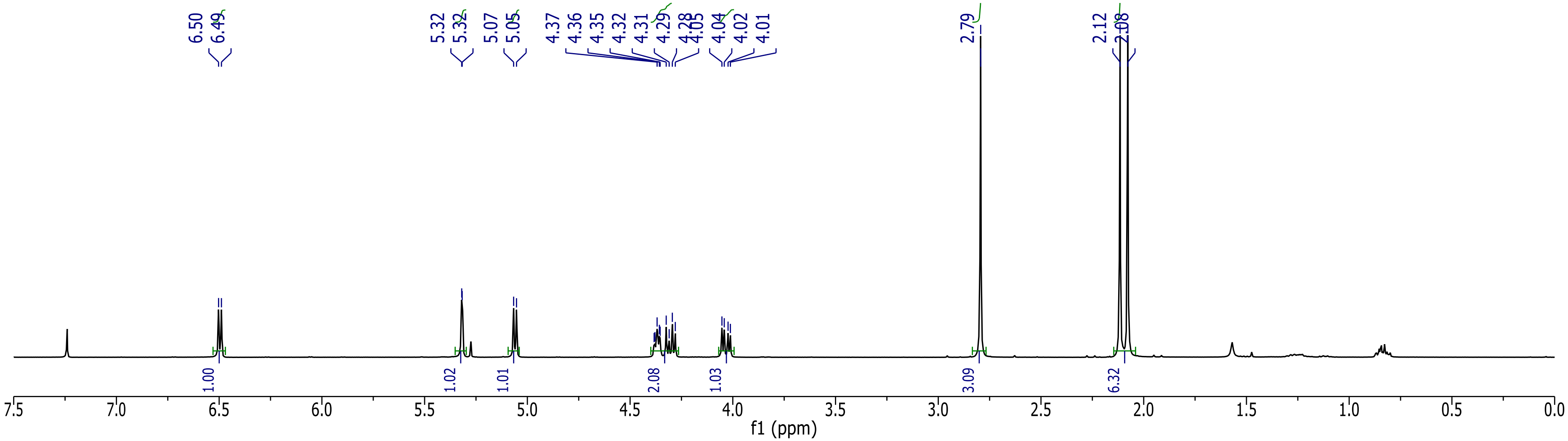
6.50
6.49
5.32
5.32
5.07
5.05
4.37
4.36
4.35
4.32
4.31
4.29
4.28
4.05
4.04
4.02
4.01
2.79
2.12
2.08
1.00
1.02
1.01
2.08
1.03
3.09
6.32
7.5
7.0
6.5
6.0
5.5
5.0
4.5
4.0
3.5
3.0
2.5
2.0
1.5
1.0
0.5
0.0
f1 (ppm)

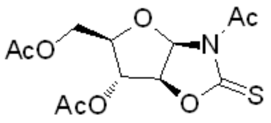
AcO
O
N
Ac
AcO
O
S

4

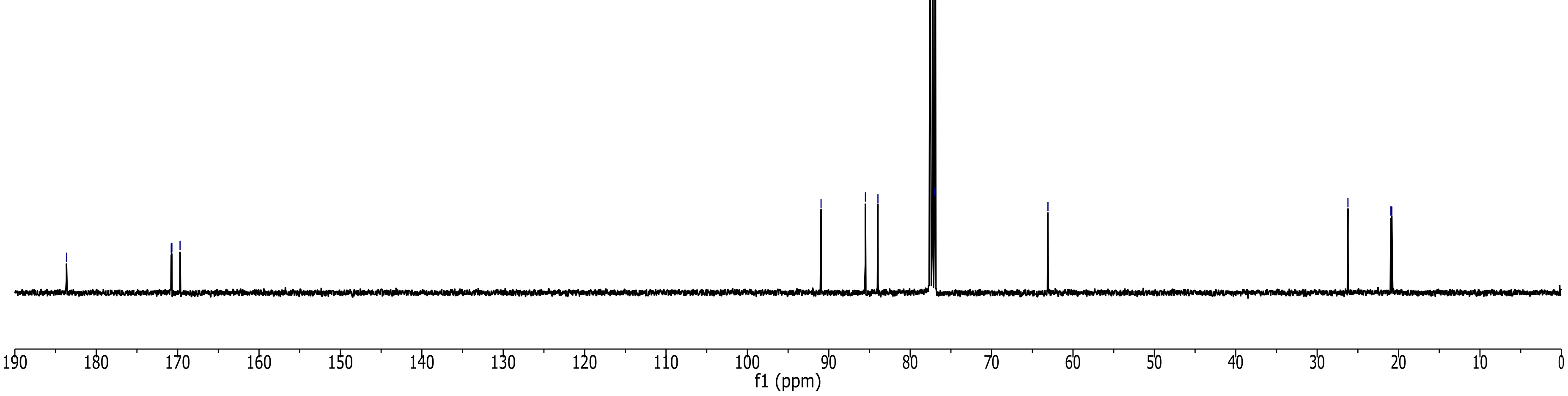
183.66
170.79
170.71
169.70
90.95
85.50
83.96
77.03
63.08
26.22
20.94
20.83
190
180
170
160
150
140
130
120
110
100
90
80
70
60
50
40
30
20
10
0
f1 (ppm)

Figure S1. ***N*-acetyl-4,5-dihydro(3′,5′-di-*O*-acetyl-1′,2′-dideoxy-*β*-*D*-arabinofuranoso)-[1,2-*d*]-oxazolidine-2-thione (4). ^{1}H NMR (400 MHz, $CDCl_3$) and ^{13}C NMR (100 MHz, $CDCl_3$) spectrums.**

Acquisition Parameter

Source Type	ESI	Ion Polarity	Positive	Set Nebulizer	0.6 Bar
Focus	Not active	Set Capillary	4500 V	Set Dry Heater	200 ℃
Scan Begin	50 m/z	Set End Plate Offset	-500 V	Set Dry Gas	7.0 l/min
Scan End	3000 m/z	Set Collision Cell RF	1000.0 Vpp	Set Divert Valve	Waste

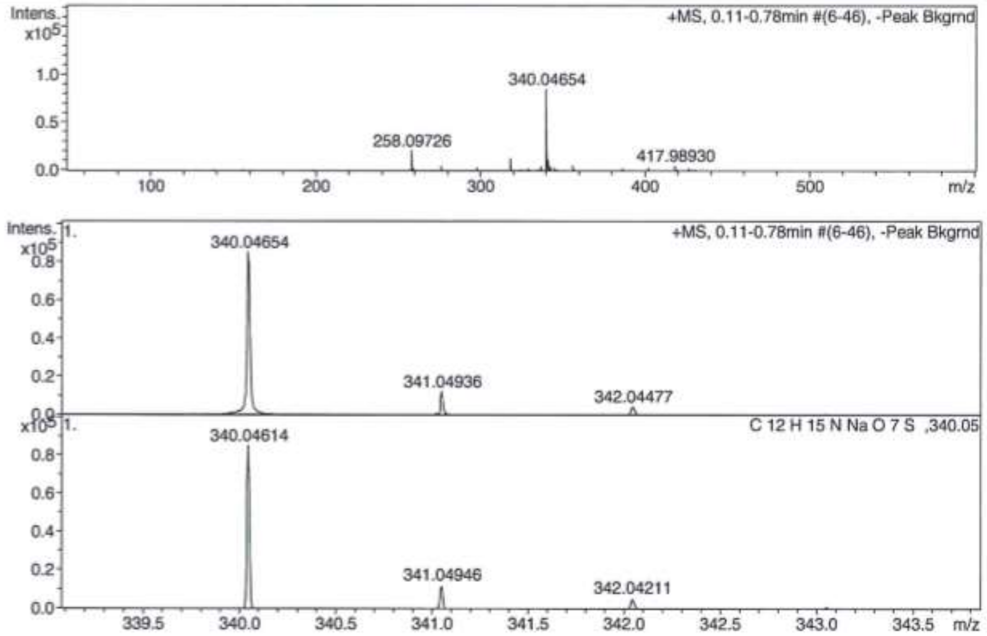

Meas. m/z	#	Formula	m/z	err [ppm]	mSigma	rdb	e⁻ Conf	N-Rule
318.06432	1	C 12 H 16 N O 7 S	318.06420	-0.4	34.9	5.5	even	ok
340.04654	1	C 12 H 15 N Na O 7 S	340.04614	-1.2	9.0	5.5	even	ok

Figure S2. ***N*-acetyl-4,5-dihydro(3′,5′-di-*O*-acetyl-1′,2′-dideoxy-*β*-*D*-arabinofuranoso)-[1,2-*d*]-oxazolidine-2-thione (4). HRMS (ESI).**

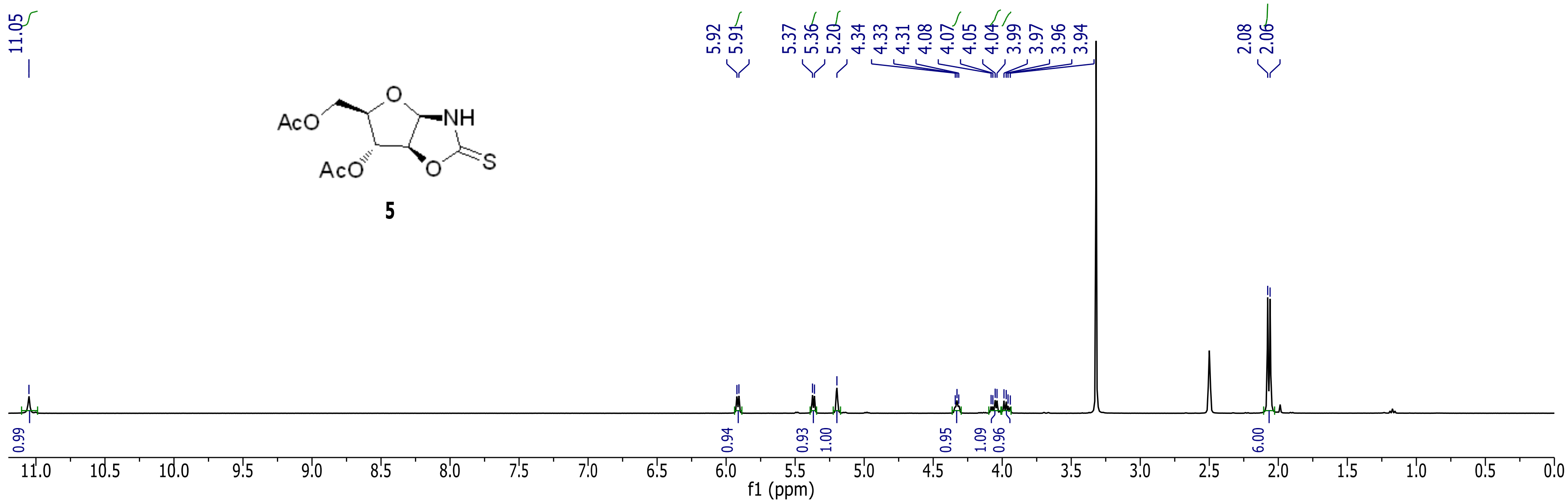
11.05
5.92
5.91
5.37
5.36
5.20
4.34
4.33
4.31
4.08
4.07
4.05
4.04
3.99
3.97
3.96
3.94
2.08
2.06
AcO
O
NH
S
AcO
O
5
0.99
0.94
0.93
1.00
0.95
1.09
0.96
6.00
11.0 10.5 10.0 9.5 9.0 8.5 8.0 7.5 7.0 6.5 6.0 5.5 5.0 4.5 4.0 3.5 3.0 2.5 2.0 1.5 1.0 0.5 0.0
f1 (ppm)

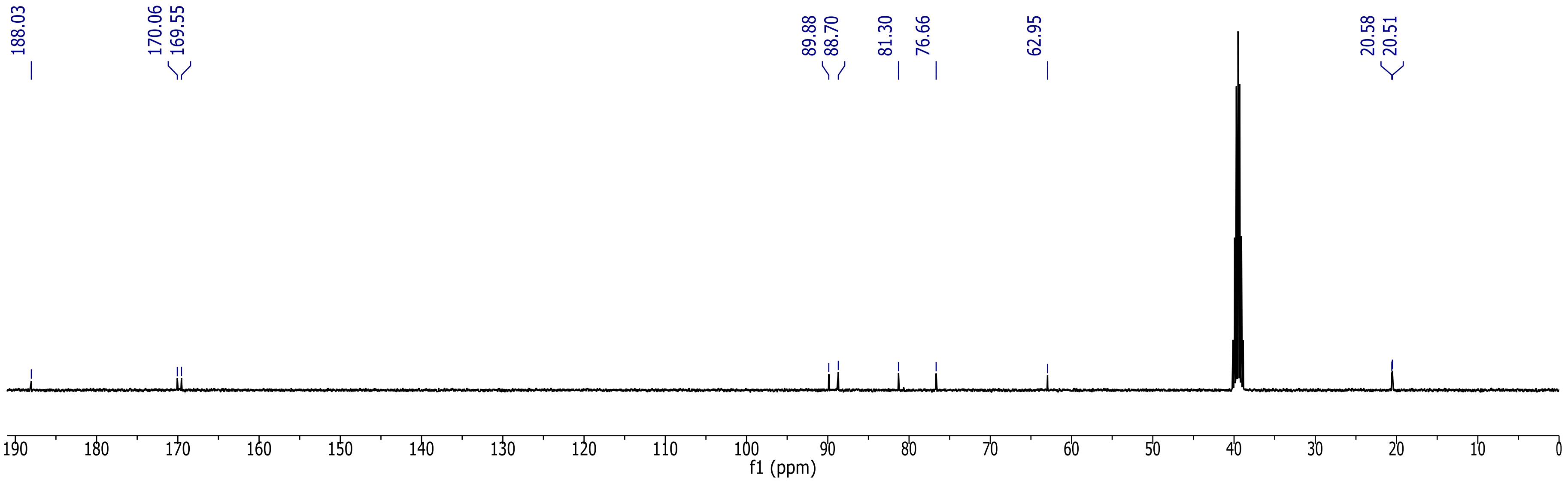
188.03
170.06
169.55
89.88
88.70
81.30
76.66
62.95
20.58
20.51
190 180 170 160 150 140 130 120 110 100 90 80 70 60 50 40 30 20 10 0
f1 (ppm)

Figure S3. 4,5-Dihydro(3′,5′-di-*O*-acetyl-1′,2′-dideoxy-*β*-*D*-arabinofuranoso)-[1,2-*d*]-oxazolidine-2-thione (5). ^{1}H NMR (400 MHz, DMSO-d_6) and ^{13}C NMR (100 MHz, DMSO-d_6) spectrums.

Acquisition Parameter

Source Type	ESI	Ion Polarity	Positive	Set Nebulizer	0.6 Bar
Focus	Not active	Set Capillary	4500 V	Set Dry Heater	200 ℃
Scan Begin	50 m/z	Set End Plate Offset	-500 V	Set Dry Gas	7.0 l/min
Scan End	3000 m/z	Set Collision Cell RF	1000.0 Vpp	Set Divert Valve	Waste

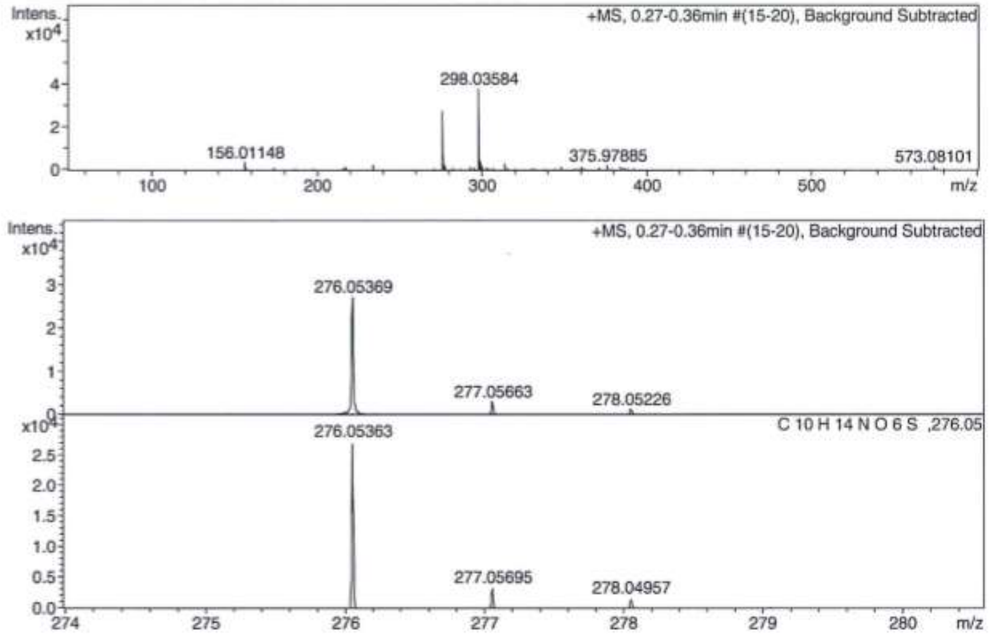

Meas. m/z	#	Formula	m/z	err [ppm]	mSigma	rdb	e⁻ Conf	N-Rule
276.05369	1	C 10 H 14 N O 6 S	276.05363	-0.2	10.2	4.5	even	ok
298.03584	1	C 10 H 13 N Na O 6 S	298.03558	-0.9	8.3	4.5	even	ok

Figure S4. 4,5-Dihydro(3′,5′-di-*O*-acetyl-1′,2′-dideoxy-*β*-*D*-arabinofuranoso)-[1,2-*d*]-oxazolidine-2-thione (5). HRMS (ESI).

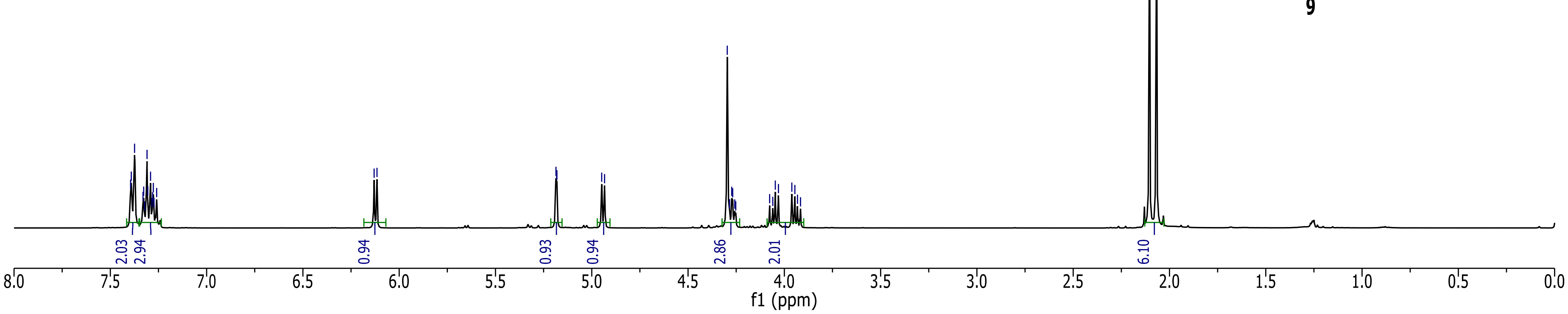
7.39
7.39
7.37
7.33
7.33
7.32
7.31
7.31
7.29
7.28
7.28
7.28
7.27
7.26
6.13
6.12
5.19
5.18
5.18
4.95
4.93
4.30
4.28
4.27
4.27
4.26
4.25
4.08
4.06
4.05
4.03
3.96
3.95
3.93
3.92
2.10
2.07
2.03
2.94
0.94
0.93
0.94
2.86
2.01
6.10
8.0 7.5 7.0 6.5 6.0 5.5 5.0 4.5 4.0 3.5 3.0 2.5 2.0 1.5 1.0 0.5 0.0
f1 (ppm)

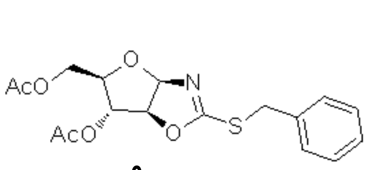
AcO
O
N
AcO
O
S
9

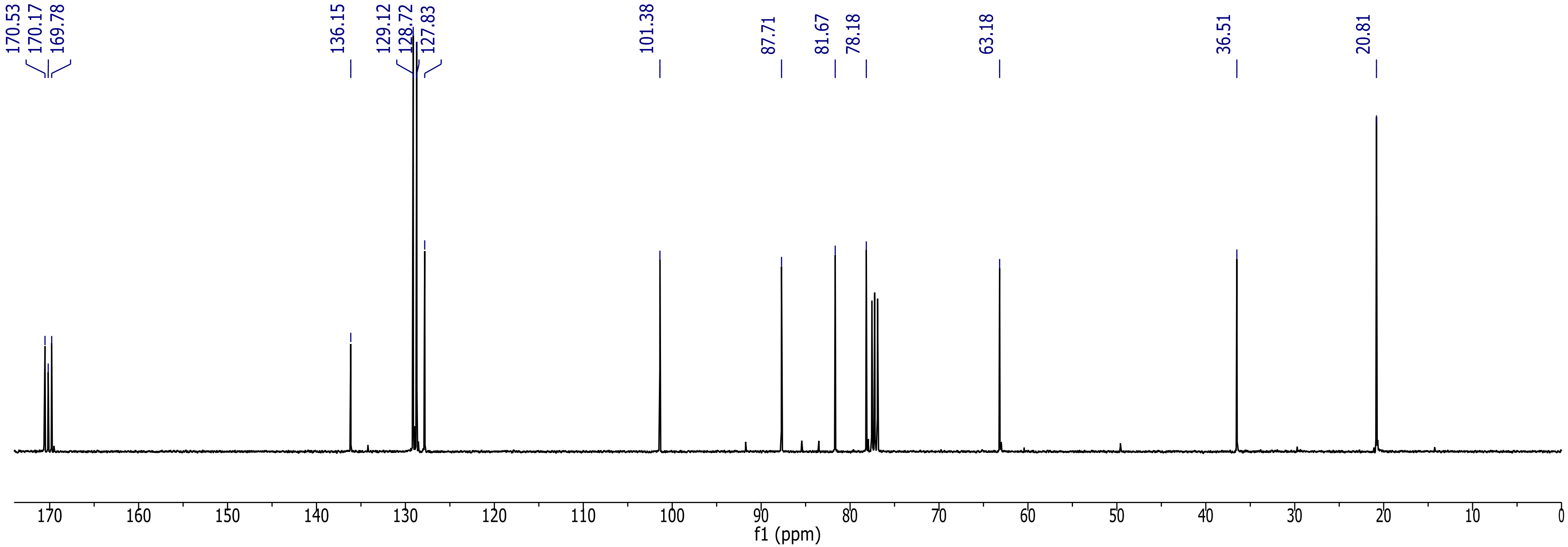
170.53
170.17
169.78
136.15
129.12
128.72
127.83
101.38
87.71
81.67
78.18
63.18
36.51
20.81
170 160 150 140 130 120 110 100 90 80 70 60 50 40 30 20 10 0
f1 (ppm)

Figure S5. 2-Benzylsulfanyl-4,5-dihydro(3′,5′-di-*O*-acetyl-1′,2′-dideoxy-*β*-*D*-arabinofuranoso)-[1,2-*d*]-oxazole (9). ^{1}H NMR (400 MHz, $CDCl_3$) and ^{13}C NMR (100 MHz, $CDCl_3$) spectrums.

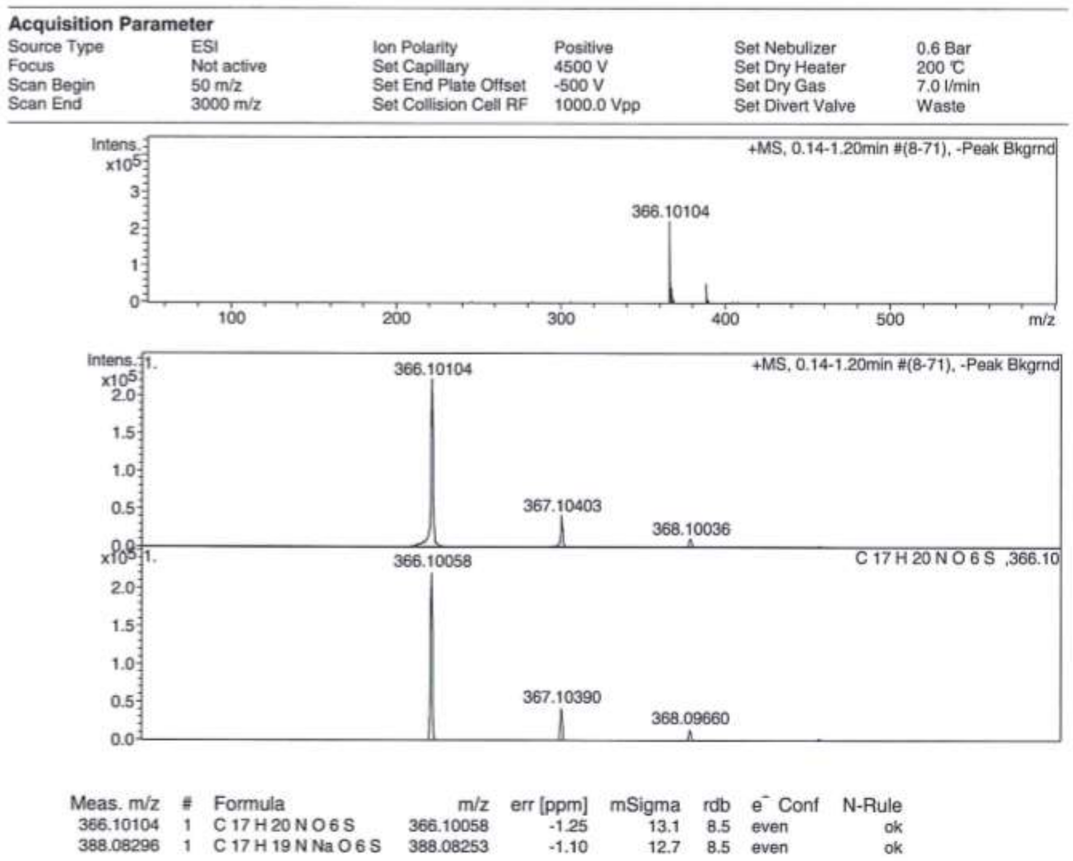

Figure S6. 2-Benzylsulfanyl-4,5-dihydro(3′,5′-di-*O*-acetyl-1′,2′-dideoxy-*β*-*D*-arabinofuranoso)-[1,2-*d*]-oxazole (9). HRMS (ESI).

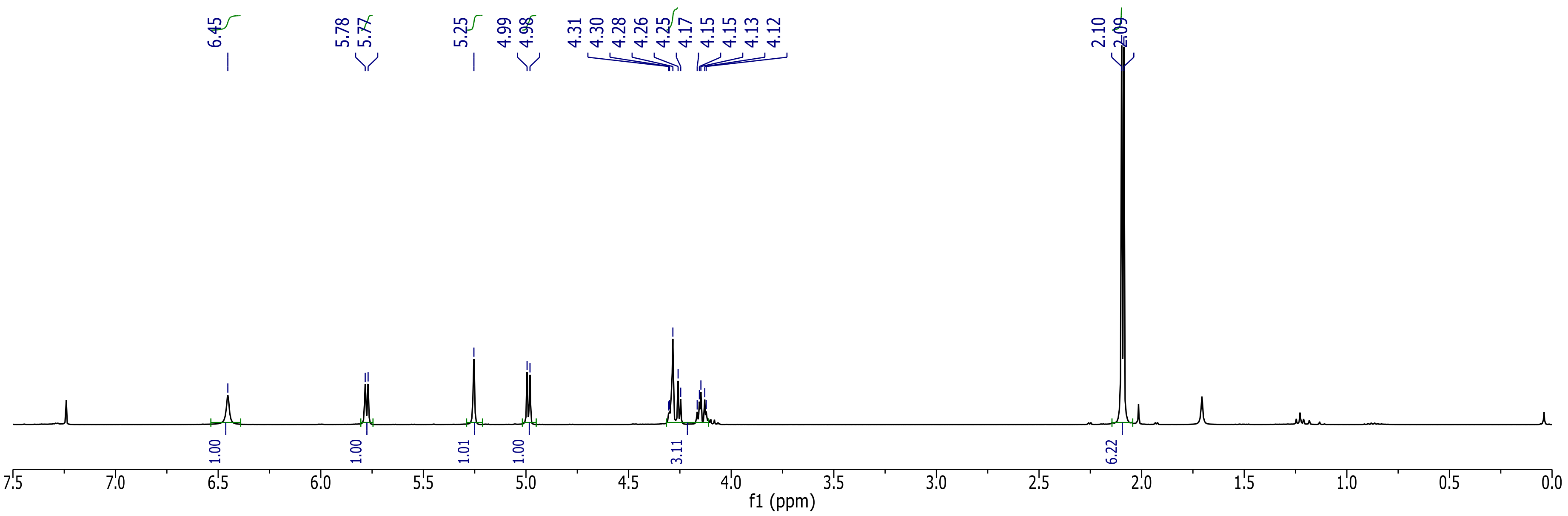

6.45
5.78
5.77
5.25
4.99
4.98
4.31
4.30
4.28
4.26
4.25
4.17
4.15
4.15
4.13
4.12
2.10
2.09
1.00
1.00
1.01
1.00
3.11
6.22
7.5
7.0
6.5
6.0
5.5
5.0
4.5
4.0
3.5
3.0
2.5
2.0
1.5
1.0
0.5
0.0
f1 (ppm)

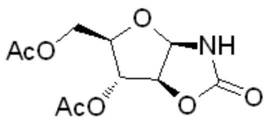

AcO
AcO
O
NH
O
O

10

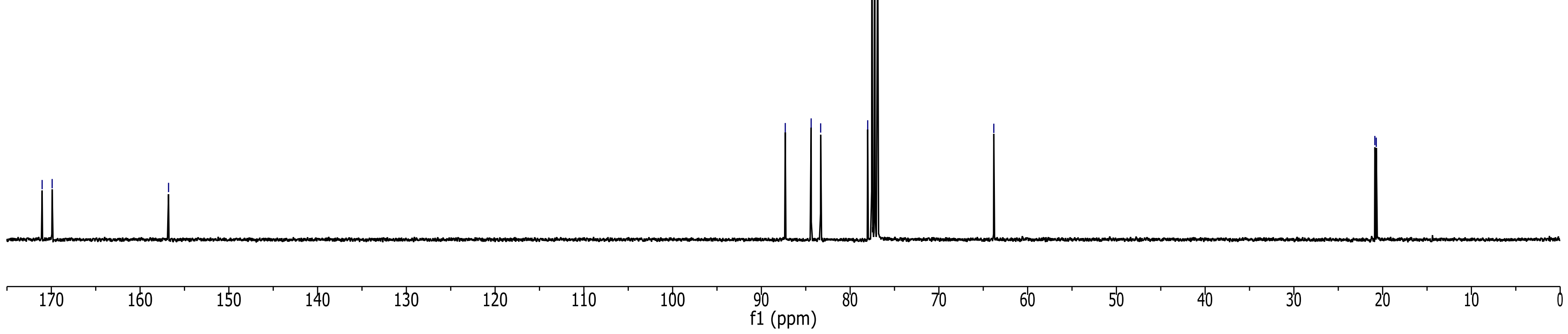

171.04
169.91
156.80
87.31
84.39
83.32
78.02
63.81
20.88
20.72
170
160
150
140
130
120
110
100
90
80
70
60
50
40
30
20
10
0
f1 (ppm)

Figure S7. 4,5-Dihydro(3′,5′-di-*O*-acetyl-1′,2′-dideoxy-*β*-*D*-arabinofuranoso)-[1,2-*d*]-oxazolidine-2-one (10). ^{1}H NMR (400 MHz, $CDCl_3$) and ^{13}C NMR (100 MHz, $CDCl_3$) spectrums.

Acquisition Parameter

Source Type	ESI	Ion Polarity	Positive	Set Nebulizer	0.6 Bar		
Focus	Not active	Set Capillary	4500 V	Set Dry Heater	200 ℃		
Scan Begin	50 m/z	Set End Plate Offset	-500 V	Set Dry Gas	7.0 l/min		
Scan End	3000 m/z	Set Collision Cell RF	1000.0 Vpp	Set Divert Valve	Waste		

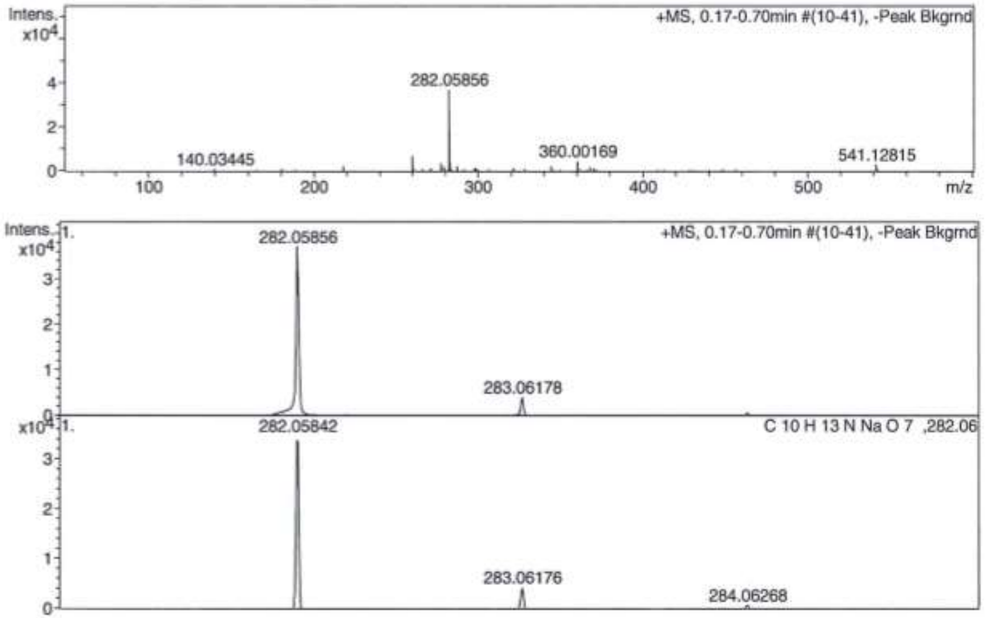

Meas. m/z	#	Formula	m/z	err [ppm]	mSigma	rdb	e⁻ Conf	N-Rule
260.07669	1	C 10 H 14 N O 7	260.07648	-0.80	65.6	4.5	even	ok
282.05856	1	C 10 H 13 N Na O 7	282.05842	-0.47	11.6	4.5	even	ok

Figure S8. 4,5-Dihydro(3′,5′-di-*O*-acetyl-1′,2′-dideoxy-*β*-*D*-arabinofuranoso)-[1,2-*d*]-oxazolidine-2-one (10). HRMS (ESI).

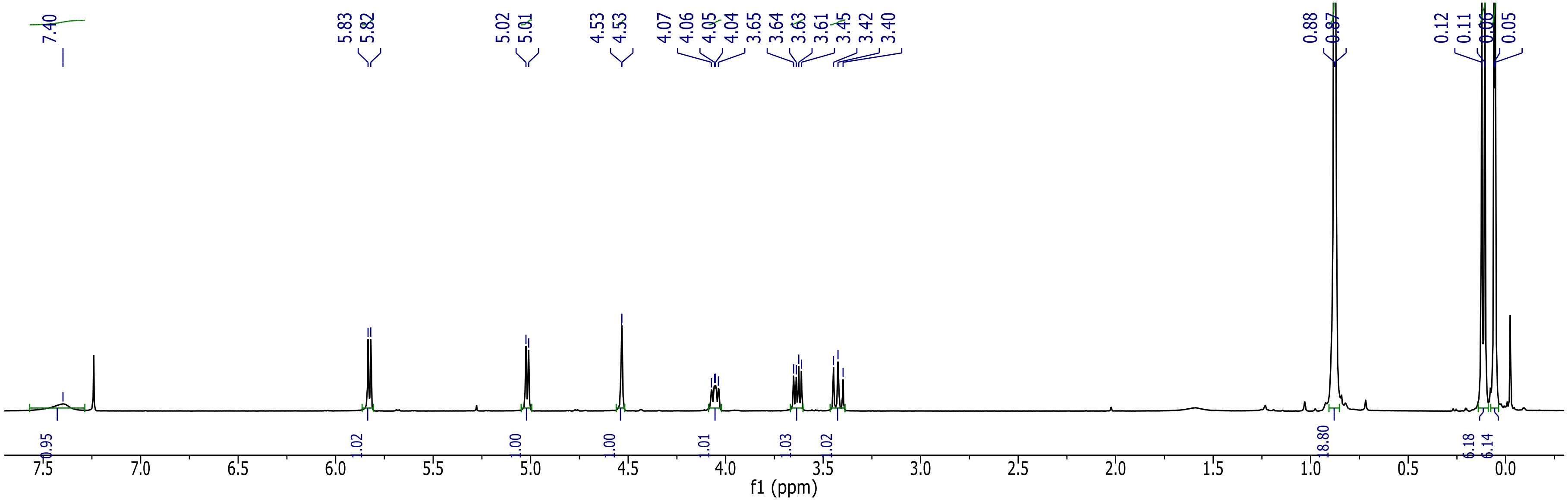
7.40
5.83
5.82
5.02
5.01
4.53
4.53
4.07
4.06
4.05
4.04
3.65
3.64
3.63
3.61
3.45
3.42
3.40
0.88
0.87
0.12
0.11
0.06
0.05
0.95
1.02
1.00
1.00
1.01
1.03
1.02
18.80
6.18
6.14
7.5
7.0
6.5
6.0
5.5
5.0
4.5
4.0
3.5
3.0
2.5
2.0
1.5
1.0
0.5
0.0
f1 (ppm)

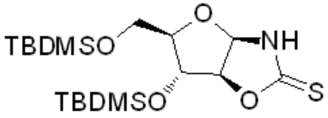
TBDMSO
TBDMSO
O
NH
O
S

6

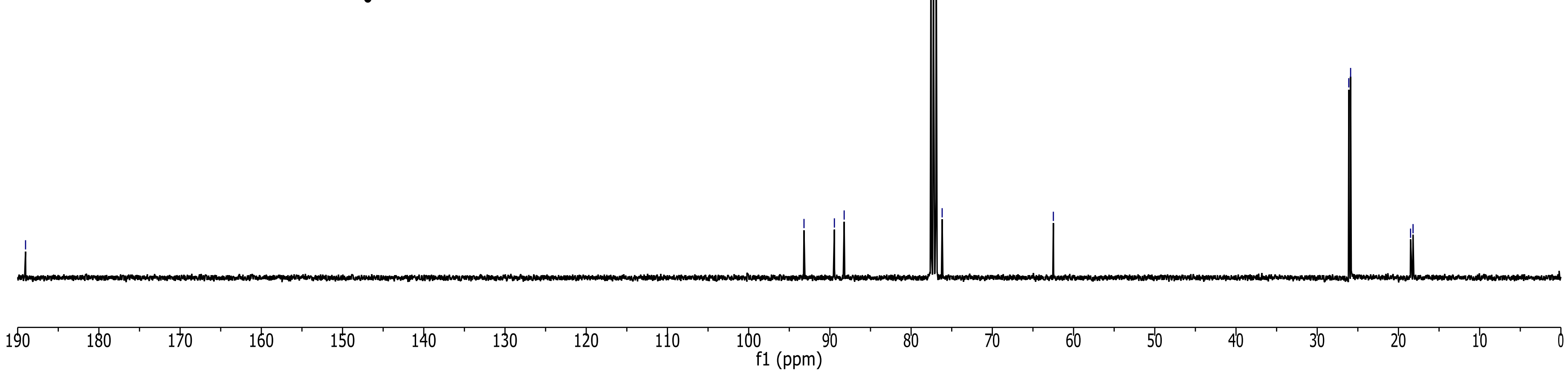
189.03
93.19
89.45
88.24
76.18
62.49
26.11
25.89
18.51
18.20
190
180
170
160
150
140
130
120
110
100
90
80
70
60
50
40
30
20
10
0
f1 (ppm)

Figure S9. **4,5-Dihydro(3′,5′-di-*O*-*tert*-butyldimethylsilyl-1′,2′-dideoxy-*β*-*D*-arabinofuranoso)-[1,2-*d*]-oxazolidine-2-thione (6). ^{1}H NMR (400 MHz, $CDCl_3$) and ^{13}C NMR (100 MHz, $CDCl_3$) spectrums.**

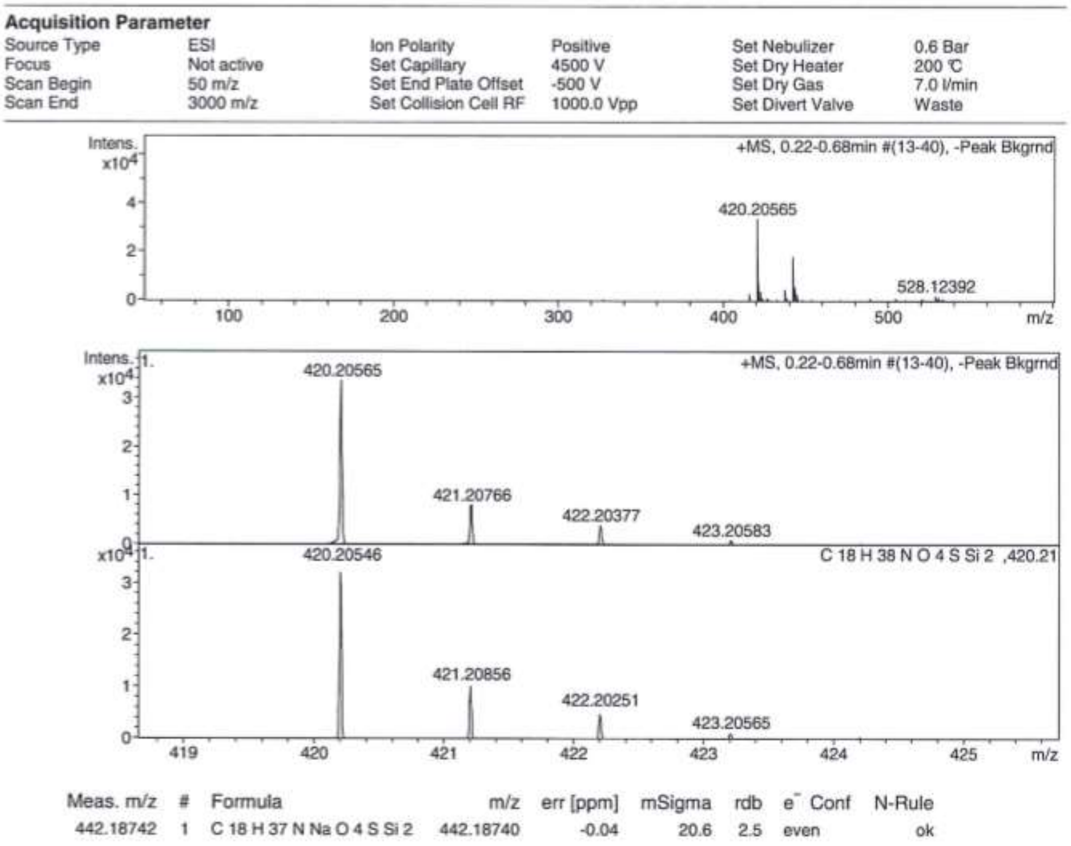

Figure S10. **4,5-Dihydro(3′,5′-di-*O*-*tert*-butyldimethylsilyl-1′,2′-dideoxy-*β*-*D*-arabinofuranoso)-[1,2-*d*]-oxazolidine-2-thione (6). HRMS (ESI).**

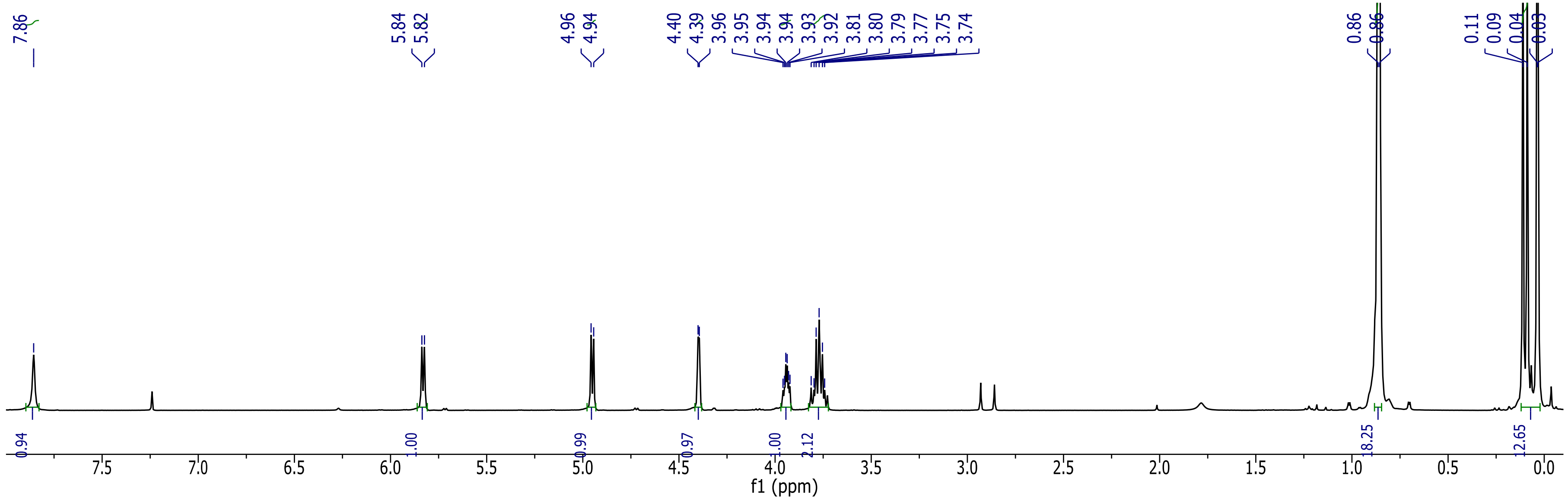

7.86
5.84 5.82
4.96 4.94
4.40 4.39 3.96 3.95 3.94 3.94 3.93 3.92 3.81 3.80 3.79 3.77 3.75 3.74
0.86 0.86
0.11 0.09 0.04 0.03
0.94
1.00
0.99
0.97
1.00
2.12
18.25
12.65
7.5 7.0 6.5 6.0 5.5 5.0 4.5 4.0 3.5 3.0 2.5 2.0 1.5 1.0 0.5 0.0
f1 (ppm)

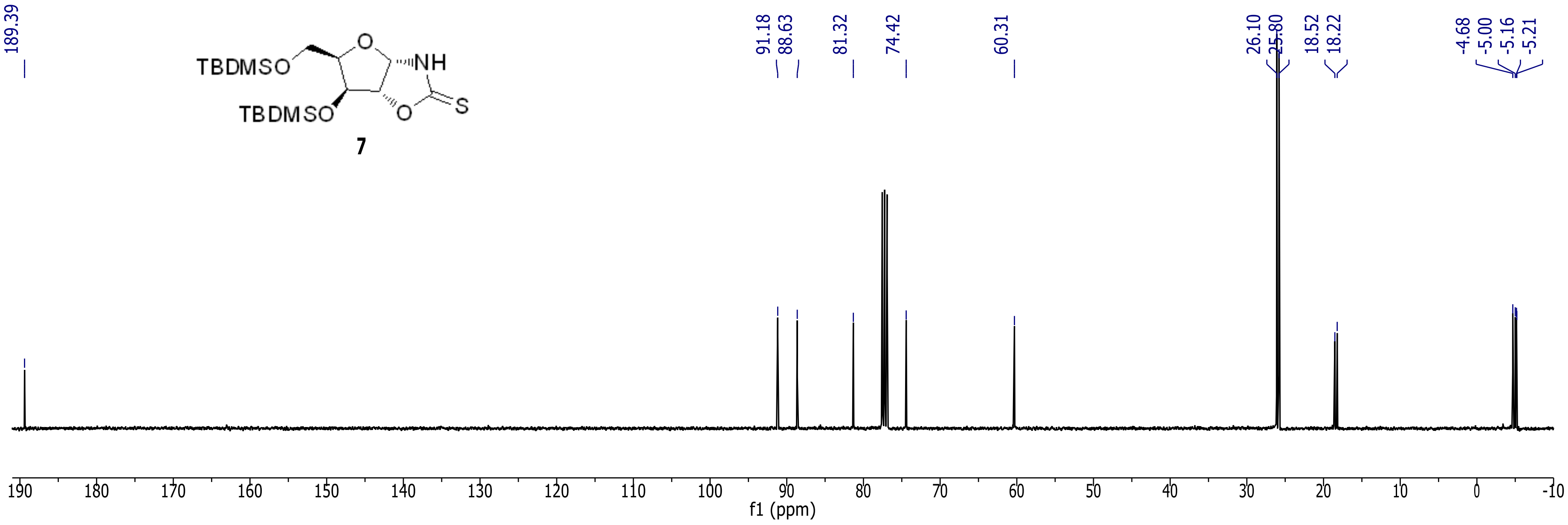

189.39
91.18 88.63 81.32 74.42 60.31
26.10 25.80 18.52 18.22
-4.68 -5.00 -5.16 -5.21
TBDMSO
TBDMSO
O
NH
O
S
7
190 180 170 160 150 140 130 120 110 100 90 80 70 60 50 40 30 20 10 0 -10
f1 (ppm)

Figure S11. 4,5-Dihydro(3′,5′-di-*O*-*tert*-butyldimethylsilyl-1′,2′-dideoxy-*α*-*D*-xylofuranoso)-[1,2-*d*]-oxazolidine-2-thione (7). ^{1}H NMR (400 MHz, $CDCl_3$) and ^{13}C NMR (100 MHz, $CDCl_3$) spectrums.

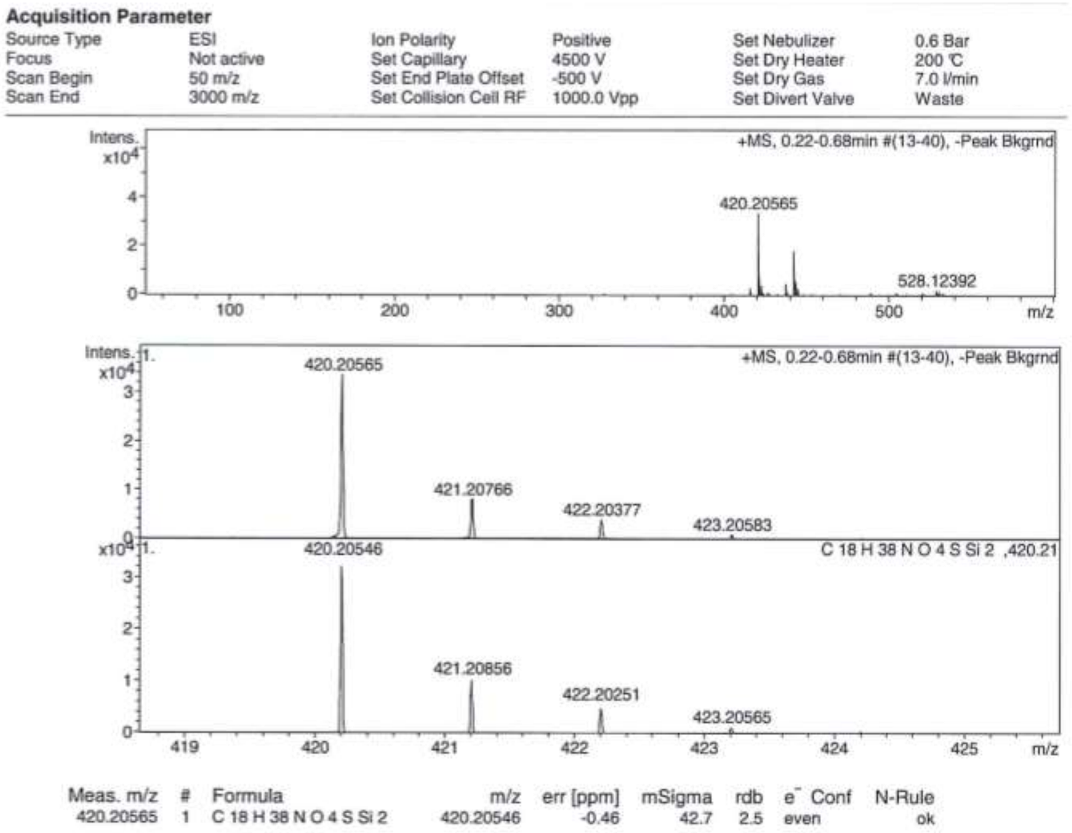

Figure S12. 4,5-Dihydro(3′,5′-di-*O*-*tert*-butyldimethylsilyl-1′,2′-dideoxy-*α*-*D*-xylofuranoso)-[1,2-*d*]-oxazolidine-2-thione (7). HRMS (ESI).

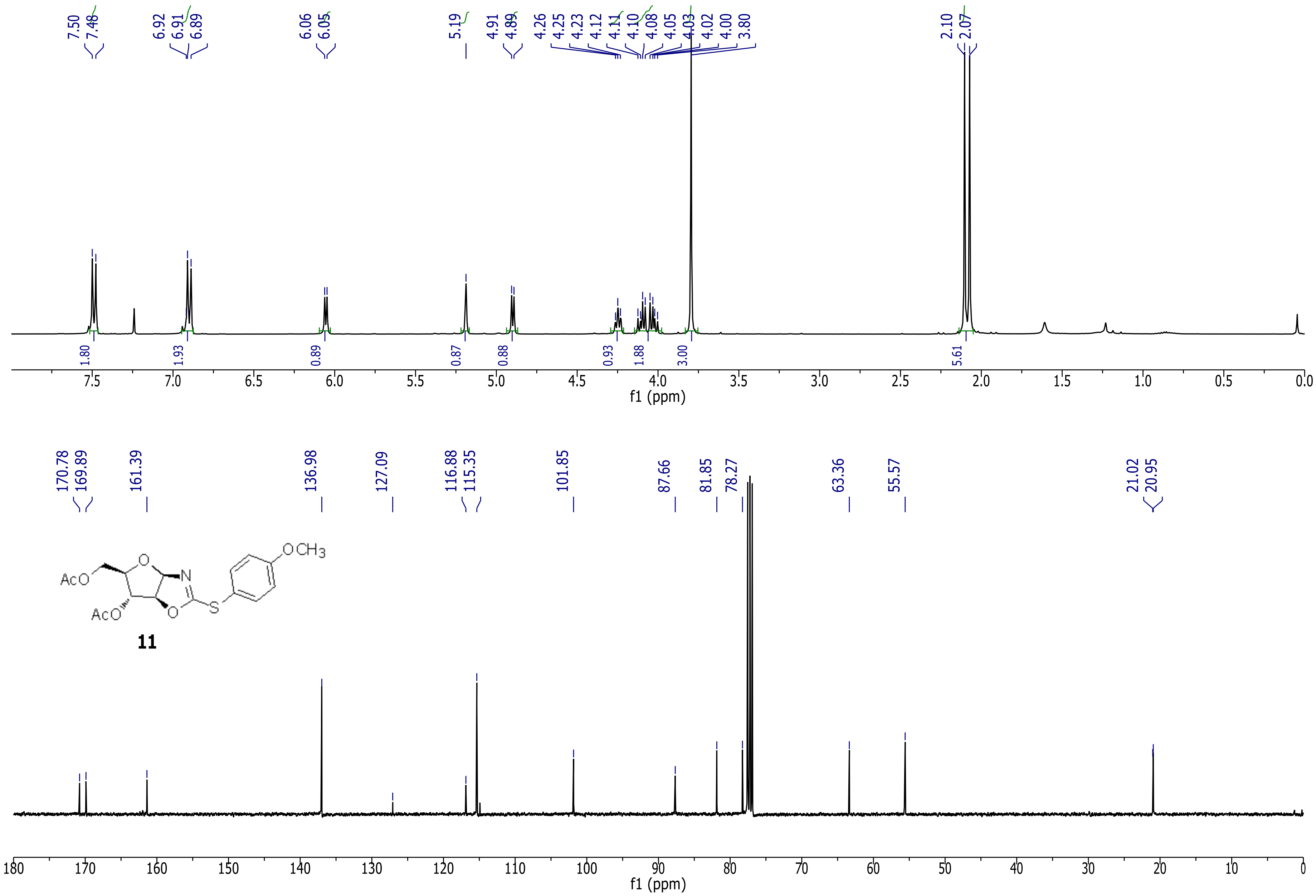
7.50
7.48
6.92
6.91
6.89
6.06
6.05
5.19
4.91
4.89
4.26
4.25
4.23
4.12
4.11
4.10
4.08
4.05
4.03
4.02
4.00
3.80
2.10
2.07
1.80
1.93
0.89
0.87
0.88
0.93
1.88
3.00
5.61
f1 (ppm)
170.78
169.89
161.39
136.98
127.09
116.88
115.35
101.85
87.66
81.85
78.27
63.36
55.57
21.02
20.95
AcO
AcO
O
N
O
S
OCH3
11
f1 (ppm)

Figure S13. 2-[(4-Methoxyphenyl)sulfanyl]-4,5-dihydro(3′,5′-di-*O*-acetyl-1′,2′-dideoxy-*β*-*D*-arabinofuranoso)-[1,2-*d*]-oxazole (11). ^{1}H NMR (400 MHz, $CDCl_3$) and ^{13}C NMR (100 MHz, $CDCl_3$) spectrums.

Acquisition Parameter

Source Type	ESI	Ion Polarity	Positive	Set Nebulizer	0.6 Bar
Focus	Not active	Set Capillary	4500 V	Set Dry Heater	200 °C
Scan Begin	50 m/z	Set End Plate Offset	-500 V	Set Dry Gas	7.0 l/min
Scan End	3000 m/z	Set Collision Cell RF	1000.0 Vpp	Set Divert Valve	Waste

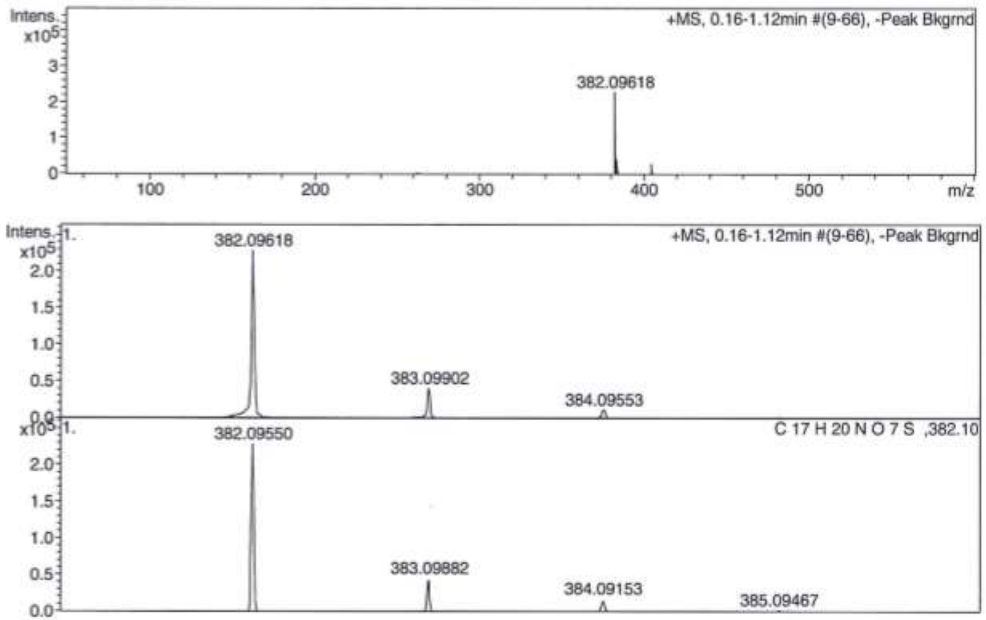

Meas. m/z	#	Formula	m/z	err [ppm]	mSigma	rdb	e⁻ Conf	N-Rule
382.09618	1	C 17 H 20 N O 7 S	382.09550	-1.79	15.4	8.5	even	ok
404.07785	1	C 17 H 19 N Na O 7 S	404.07744	-1.02	11.4	8.5	even	ok

Figure S14. 2-[(4-Methoxyphenyl)sulfanyl]-4,5-dihydro(3′,5′-di-*O*-acetyl-1′,2′-dideoxy-*β*-*D*-arabinofuranoso)-[1,2-*d*]-oxazole (11). HRMS (ESI).

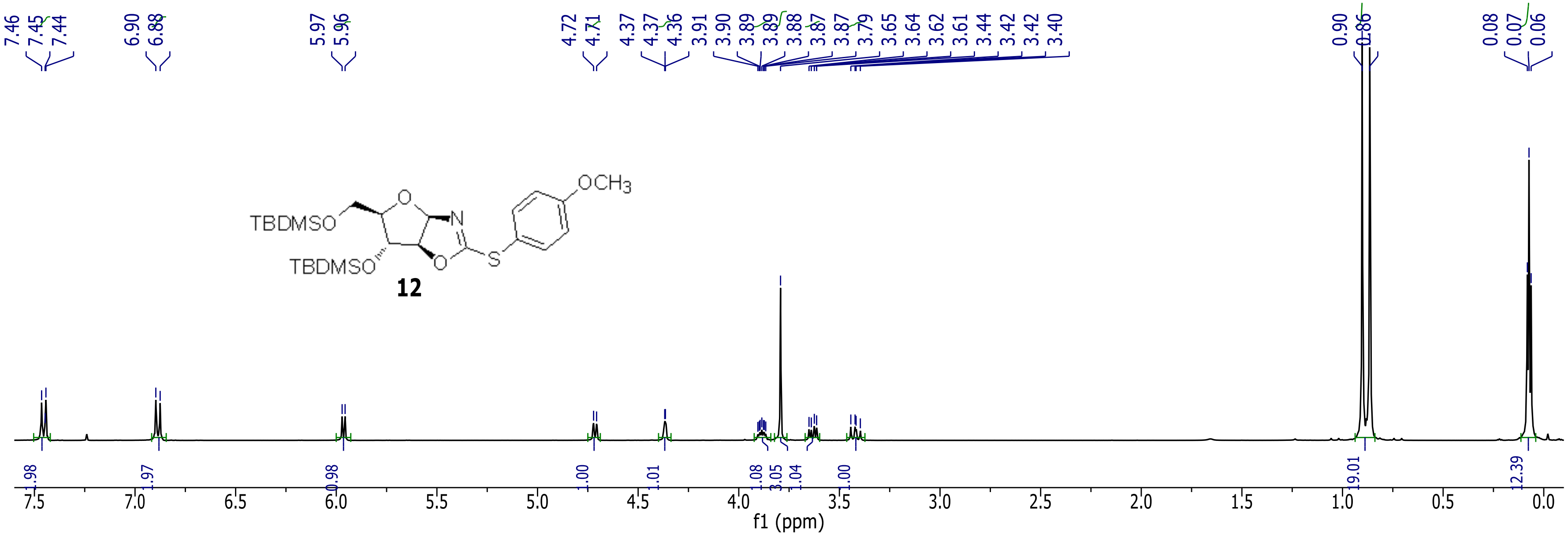
7.46 7.45 7.44 6.90 6.88 5.97 5.96 4.72 4.71 4.37 4.37 4.36 3.91 3.90 3.89 3.89 3.88 3.87 3.87 3.79 3.65 3.64 3.62 3.61 3.44 3.42 3.42 3.40 0.90 0.86 0.08 0.07 0.06
OCH3
TBDMSO
N
TBDMSO
O
S
O
12
1.98 1.97 0.98 1.00 1.01 1.08 3.05 1.04 1.00 19.01 12.39
f1 (ppm)

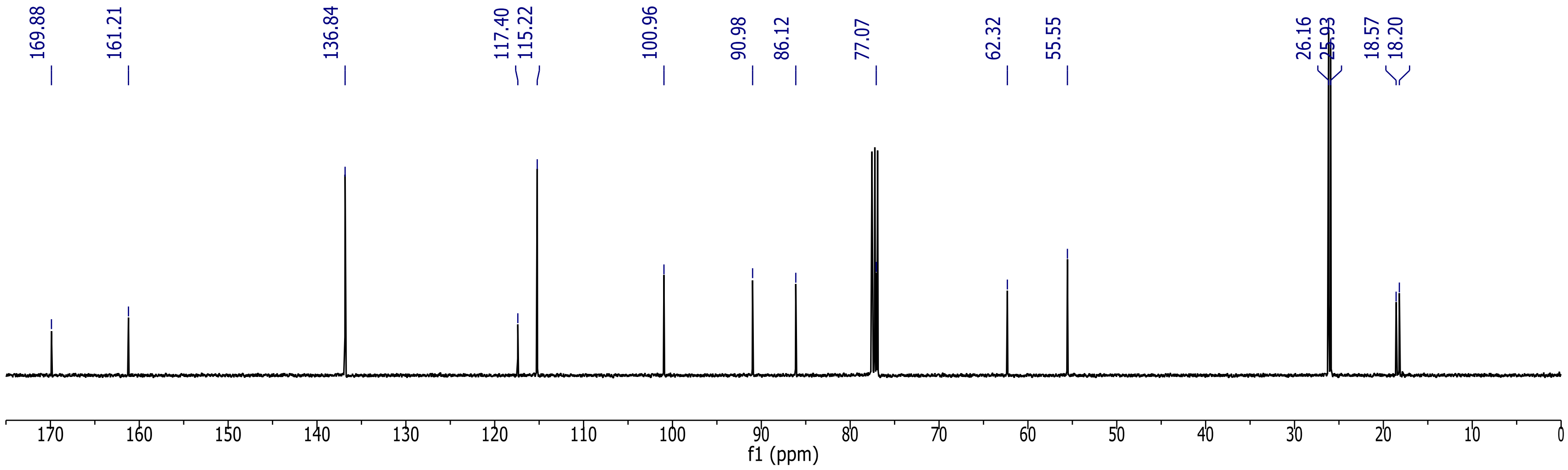
169.88 161.21 136.84 117.40 115.22 100.96 90.98 86.12 77.07 62.32 55.55 26.16 25.93 18.57 18.20
f1 (ppm)

Figure S15. 2-[(4-Methoxyphenyl)sulfanyl]-4,5-dihydro(3′,5′-di-*O*-*tert*-1′,2′-dideoxy-*β*-*D*-arabinofuranoso)-[1,2-*d*]-oxazole (12). ^{1}H NMR (400 MHz, $CDCl_3$) and ^{13}C NMR (100 MHz, $CDCl_3$) spectrums.

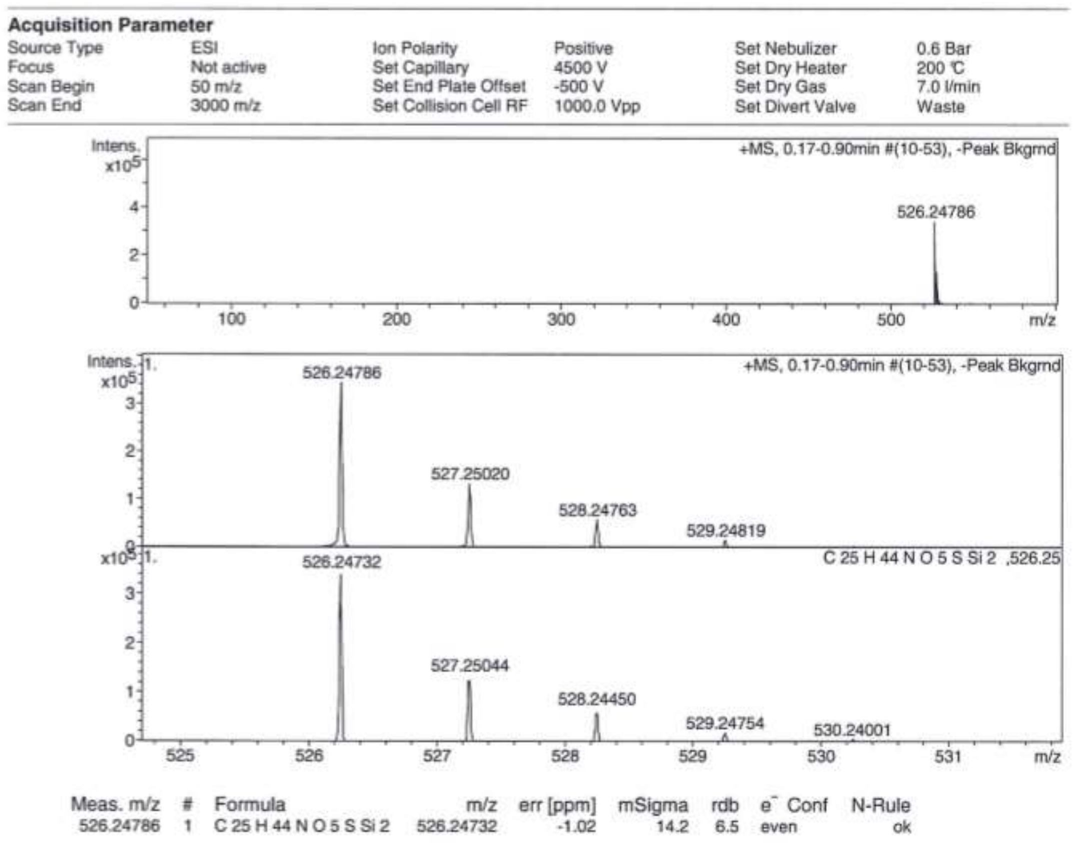

Figure S16. 2-[(4-Methoxyphenyl)sulfanyl]-4,5-dihydro(3′,5′-di-*O*-*tert*-1′,2′-dideoxy-*β*-*D*-arabinofuranoso)-[1,2-*d*]-oxazole (12). HRMS (ESI).

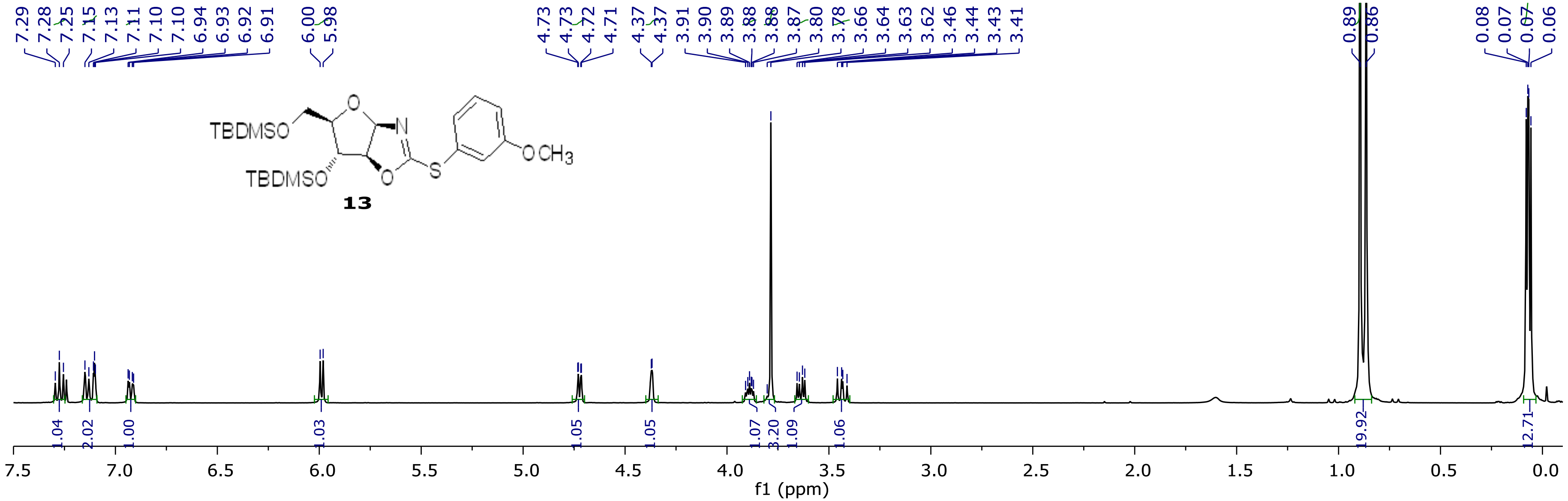
7.29 7.28 7.25 7.15 7.13 7.11 7.10 7.10 6.94 6.93 6.92 6.91
6.00 5.98
4.73 4.73 4.72 4.71
4.37 4.37
3.91 3.90 3.89 3.88 3.88 3.87 3.80 3.78 3.66 3.64 3.63 3.62 3.46 3.44 3.43 3.41
0.89 0.86
0.08 0.07 0.07 0.06
TBDMSO
TBDMSO
O
N
O
S
OCH3
13
1.04 2.02 1.00 1.03 1.05 1.05 1.07 3.20 1.09 1.06 19.92 12.71
7.5 7.0 6.5 6.0 5.5 5.0 4.5 4.0 3.5 3.0 2.5 2.0 1.5 1.0 0.5 0.0
f1 (ppm)

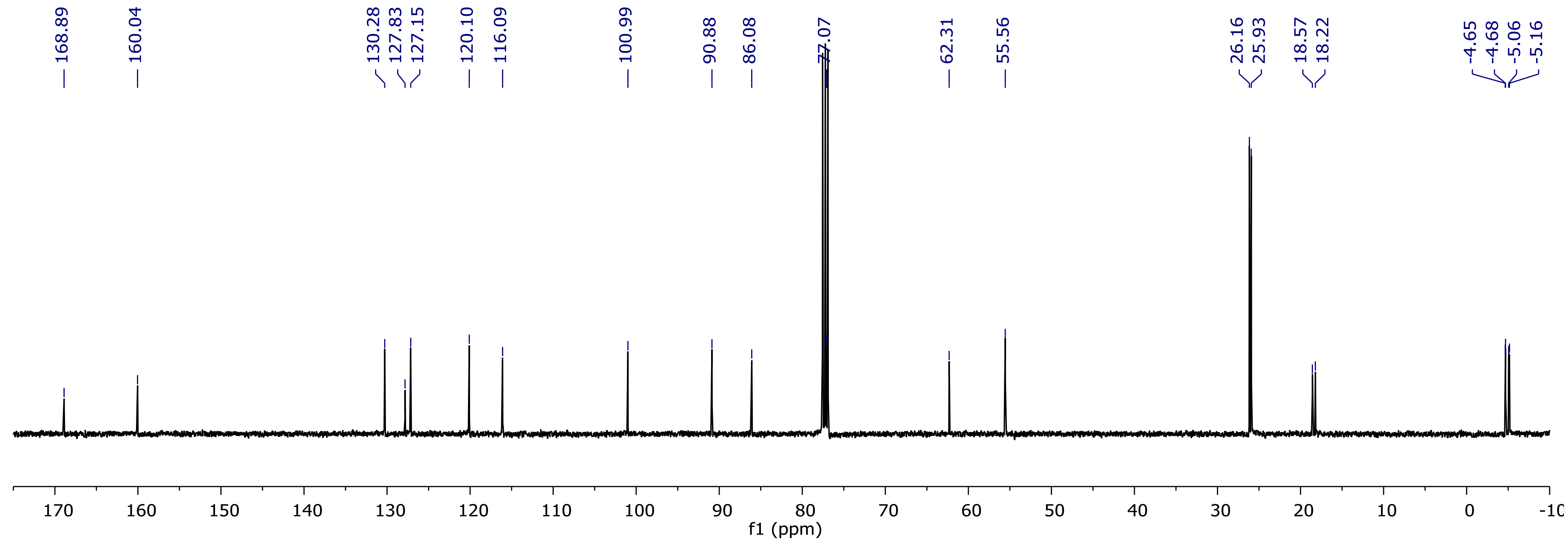
168.89
160.04
130.28 127.83 127.15
120.10
116.09
100.99
90.88
86.08
77.07
62.31
55.56
26.16 25.93
18.57 18.22
-4.65 -4.68 -5.06 -5.16
170 160 150 140 130 120 110 100 90 80 70 60 50 40 30 20 10 0 -10
f1 (ppm)

Figure S17. **2-[(3-Methoxyphenyl)sulfanyl]-4,5-dihydro(3′,5′-di-*O*-*tert*-1′,2′-dideoxy-*β*-*D*-arabinofuranoso)-[1,2-*d*]-oxazole (13). ^{1}H NMR (400 MHz, $CDCl_3$) and ^{13}C NMR (100 MHz, $CDCl_3$) spectrums.**

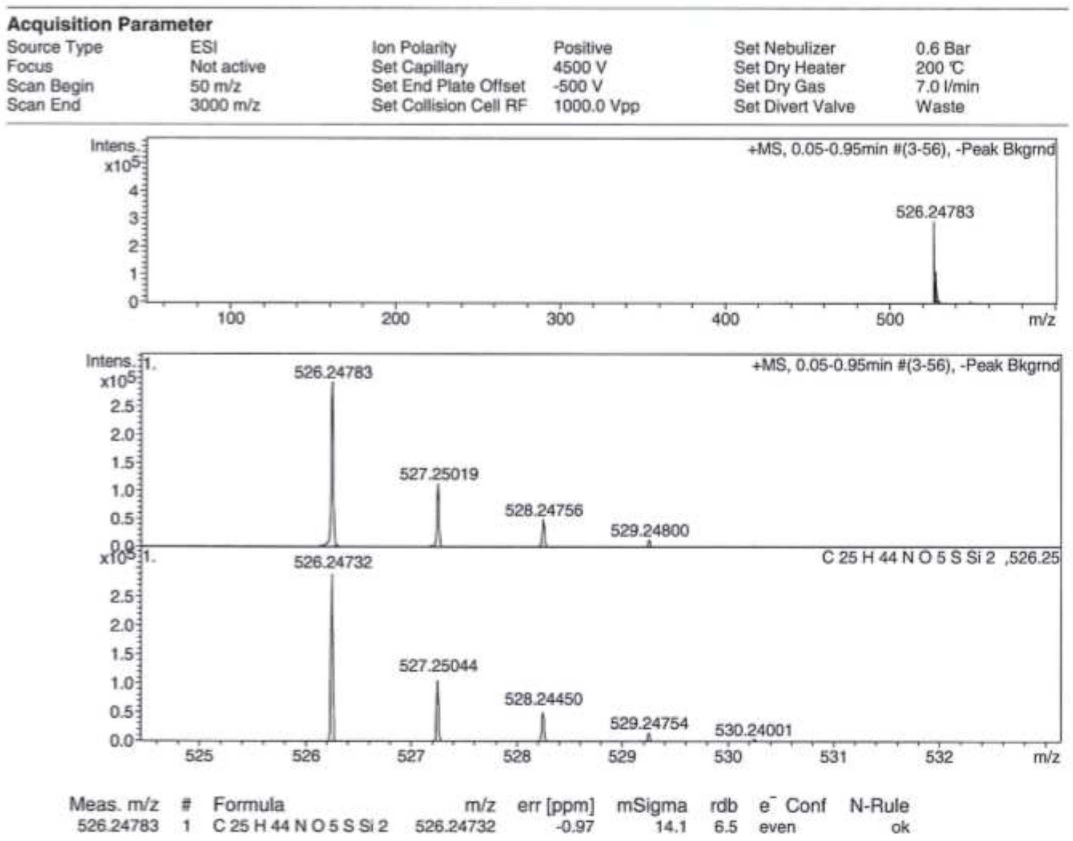

Figure S18. **2-[(3-Methoxyphenyl)sulfanyl]-4,5-dihydro(3′,5′-di-*O*-*tert*-1′,2′-dideoxy-*β*-*D*-arabinofuranoso)-[1,2-*d*]-oxazole (13). HRMS (ESI).**

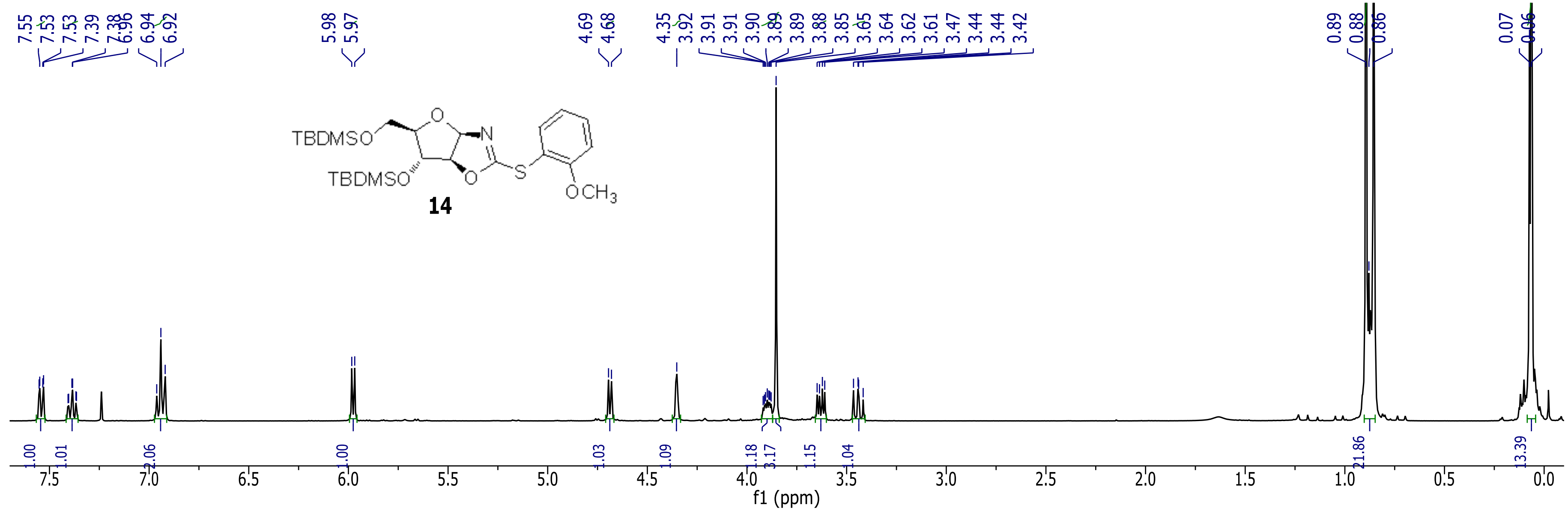
7.55
7.53
7.53
7.39
7.38
6.96
6.94
6.92
5.98
5.97
4.69
4.68
4.35
3.92
3.91
3.91
3.90
3.89
3.89
3.88
3.85
3.65
3.64
3.62
3.61
3.47
3.44
3.44
3.42
0.89
0.88
0.86
0.07
0.06
TBDMSO
TBDMSO
O
N
O
S
OCH3
14
1.00
1.01
2.06
1.00
1.03
1.09
1.18
3.17
1.15
1.04
21.86
13.39
f1 (ppm)

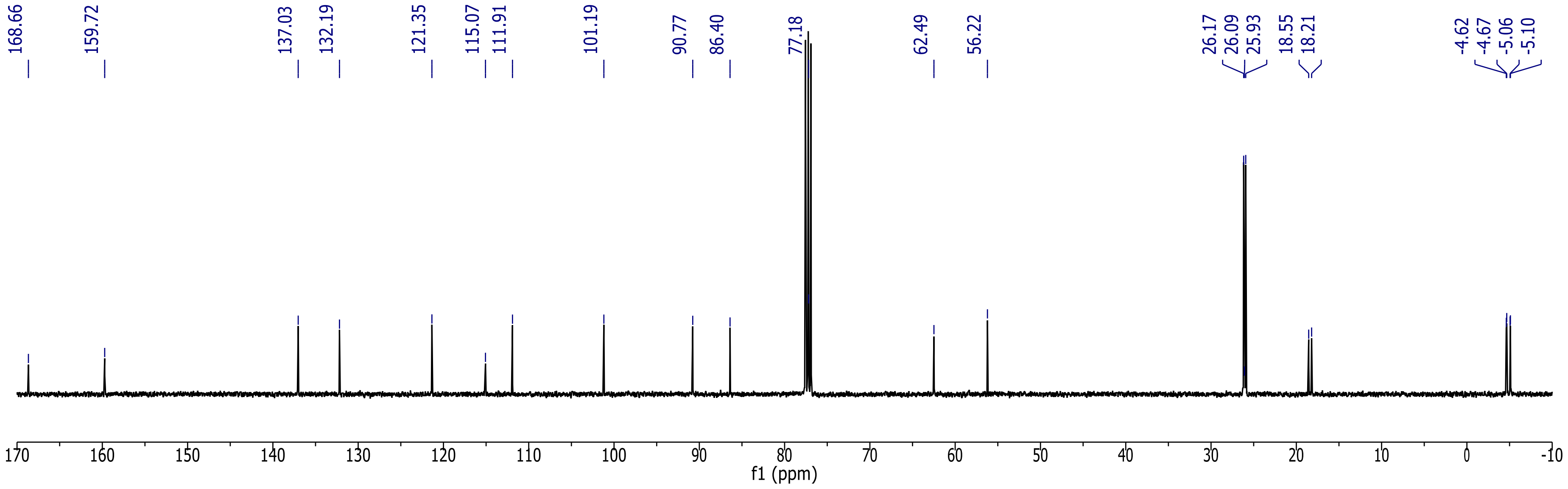
168.66
159.72
137.03
132.19
121.35
115.07
111.91
101.19
90.77
86.40
77.18
62.49
56.22
26.17
26.09
25.93
18.55
18.21
-4.62
-4.67
-5.06
-5.10
f1 (ppm)

Figure S19. 2-[(2-Methoxyphenyl)sulfanyl]-4,5-dihydro(3′,5′-di-*O*-*tert*-1′,2′-dideoxy-*β*-*D*-arabinofuranoso)-[1,2-*d*]-oxazole (14). ^{1}H NMR (400 MHz, $CDCl_3$) and ^{13}C NMR (100 MHz, $CDCl_3$) spectrums.

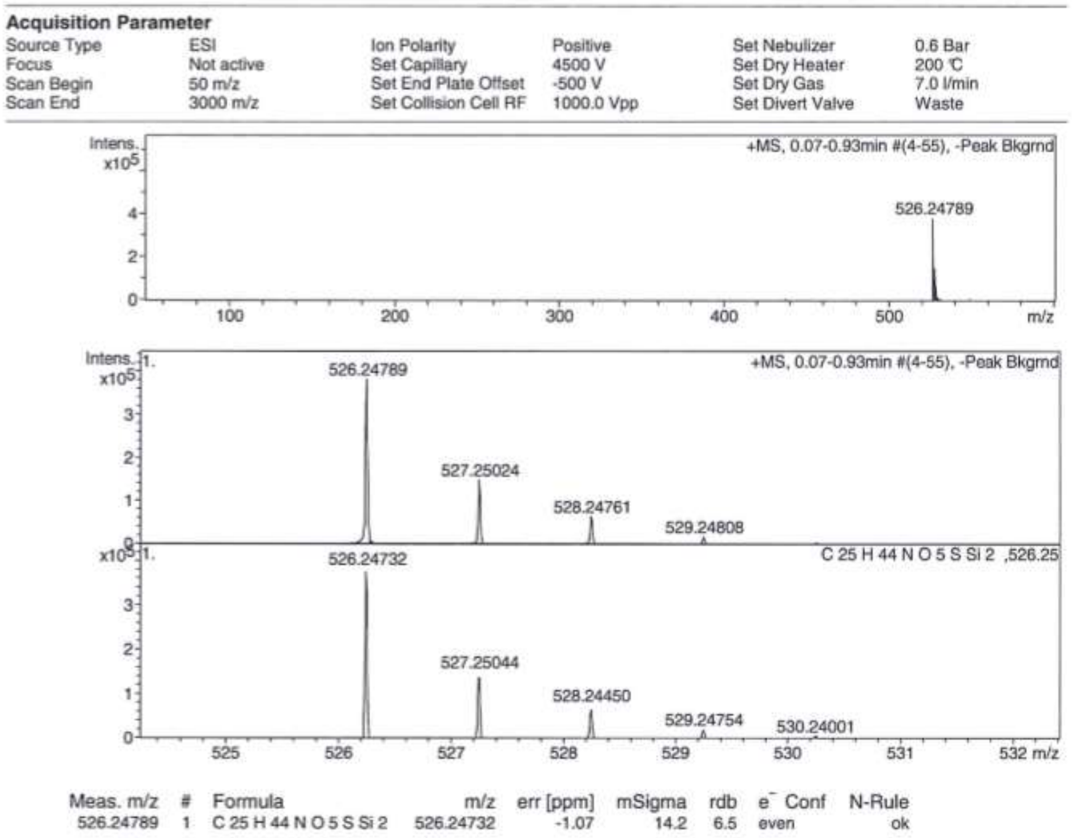

Figure S20. 2-[(2-Methoxyphenyl)sulfanyl]-4,5-dihydro(3′,5′-di-*O*-*tert*-1′,2′-dideoxy-*β*-*D*-arabinofuranoso)-[1,2-*d*]-oxazole (14). HRMS (ESI).

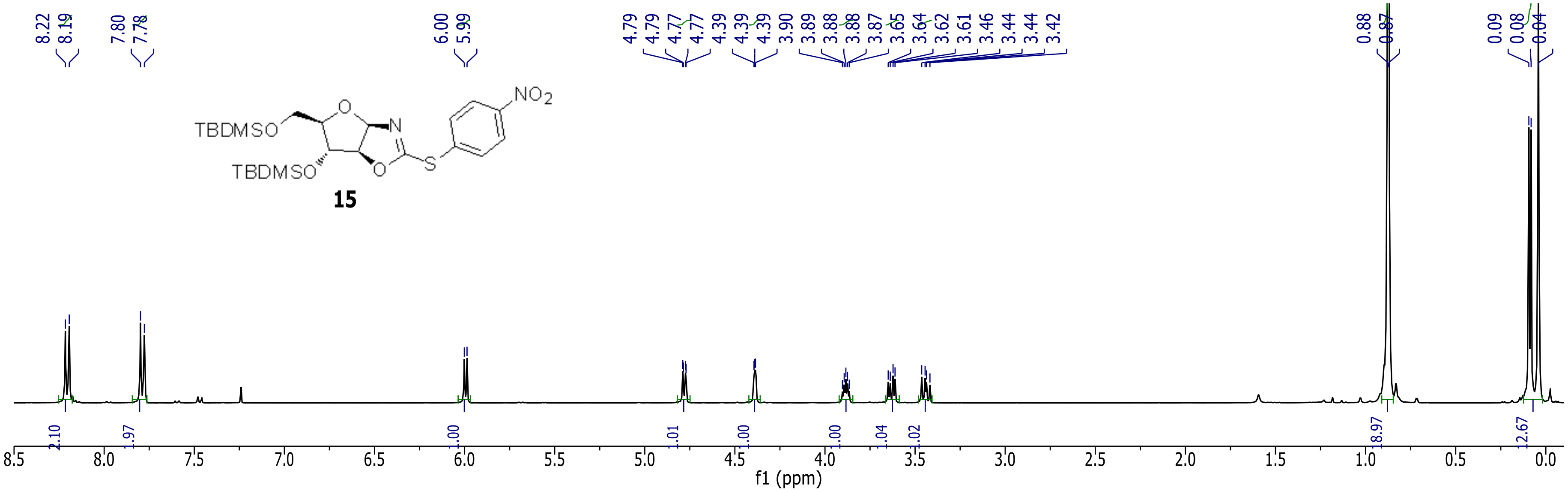
8.22
8.19
7.80
7.78
6.00
5.99
4.79
4.79
4.77
4.77
4.39
4.39
4.39
3.90
3.89
3.88
3.88
3.87
3.65
3.64
3.62
3.61
3.46
3.44
3.44
3.42
0.88
0.87
0.09
0.08
0.04
TBDMSO
TBDMSO
NO2
N
O
O
S
15
2.10
1.97
1.00
1.01
1.00
1.00
1.04
1.02
18.97
12.67
8.5
8.0
7.5
7.0
6.5
6.0
5.5
5.0
4.5
4.0
3.5
3.0
2.5
2.0
1.5
1.0
0.5
0.0
f1 (ppm)

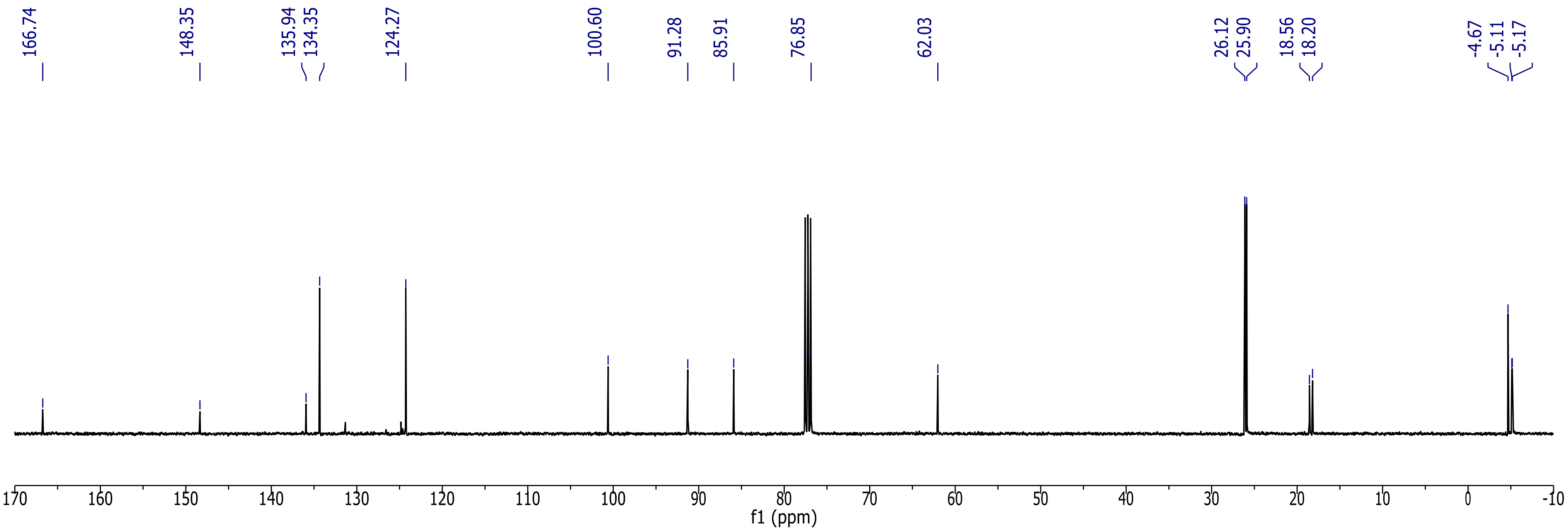
166.74
148.35
135.94
134.35
124.27
100.60
91.28
85.91
76.85
62.03
26.12
25.90
18.56
18.20
-4.67
-5.11
-5.17
170
160
150
140
130
120
110
100
90
80
70
60
50
40
30
20
10
0
-10
f1 (ppm)

Figure S21. **2-[(4-Nitrophenyl)sulfanyl]-4,5-dihydro(3′,5′-di-*O*-*tert*-1′,2′-dideoxy-*β*-*D*-arabinofuranoso)-[1,2-*d*]-oxazole (15). ^{1}H NMR (400 MHz, $CDCl_3$) and ^{13}C NMR (100 MHz, $CDCl_3$) spectrums.**

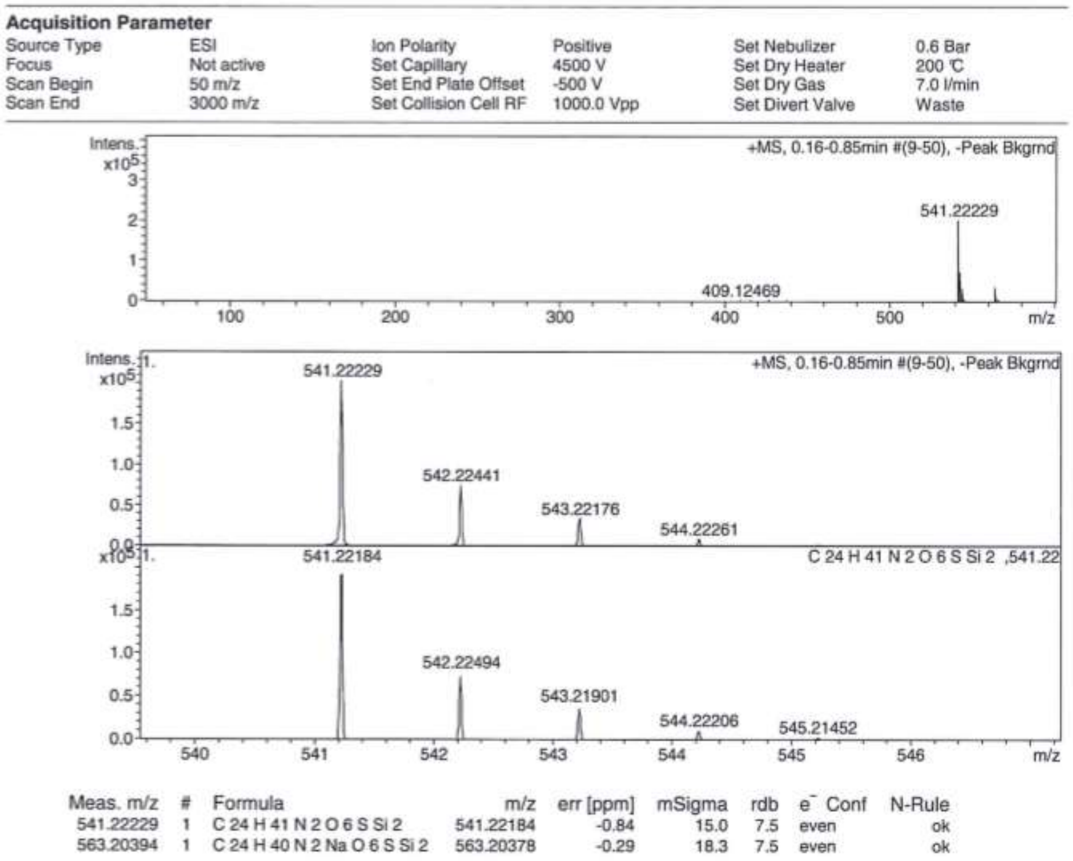

Figure S22. **2-[(4-Nitrophenyl)sulfanyl]-4,5-dihydro(3′,5′-di-*O*-*tert*-1′,2′-dideoxy-*β*-*D*-arabinofuranoso)-[1,2-*d*]-oxazole (15). HRMS (ESI).**

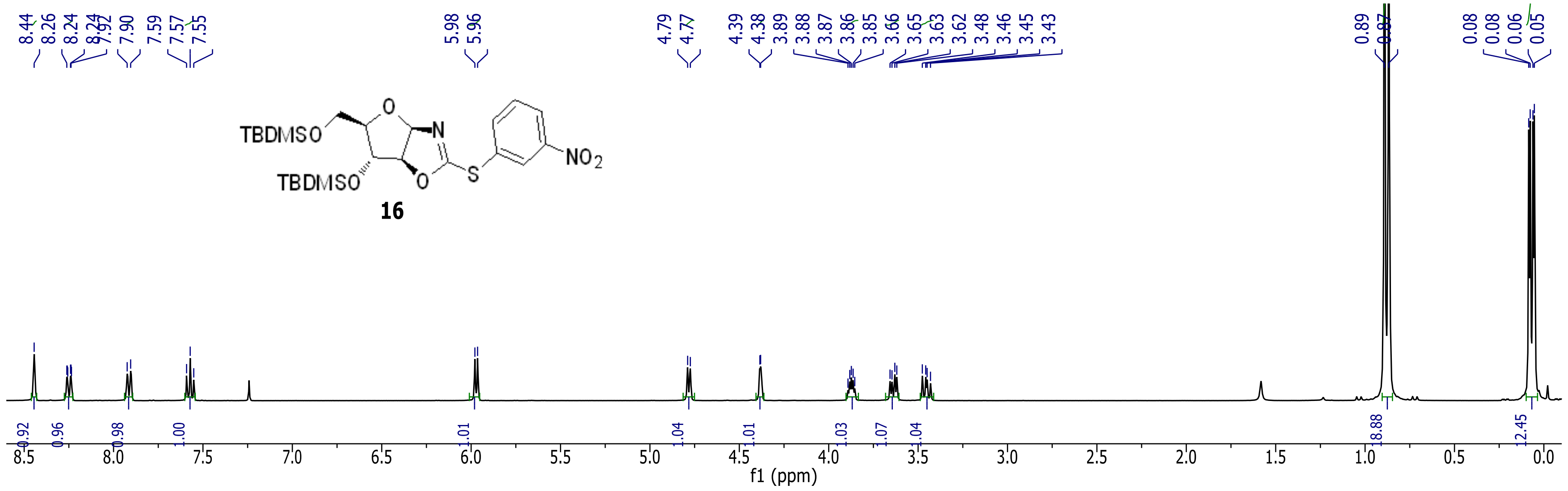
8.44
8.26
8.24
8.24
7.92
7.90
7.59
7.57
7.55
5.98
5.96
4.79
4.77
4.39
4.38
3.89
3.88
3.87
3.86
3.85
3.66
3.65
3.63
3.62
3.48
3.46
3.45
3.43
0.89
0.87
0.08
0.08
0.06
0.05
TBDMSO
TBDMSO
O
N
O
S
NO2
16
0.92
0.96
0.98
1.00
1.01
1.04
1.01
1.03
1.07
1.04
18.88
12.45
8.5
8.0
7.5
7.0
6.5
6.0
5.5
5.0
4.5
4.0
3.5
3.0
2.5
2.0
1.5
1.0
0.5
0.0
f1 (ppm)

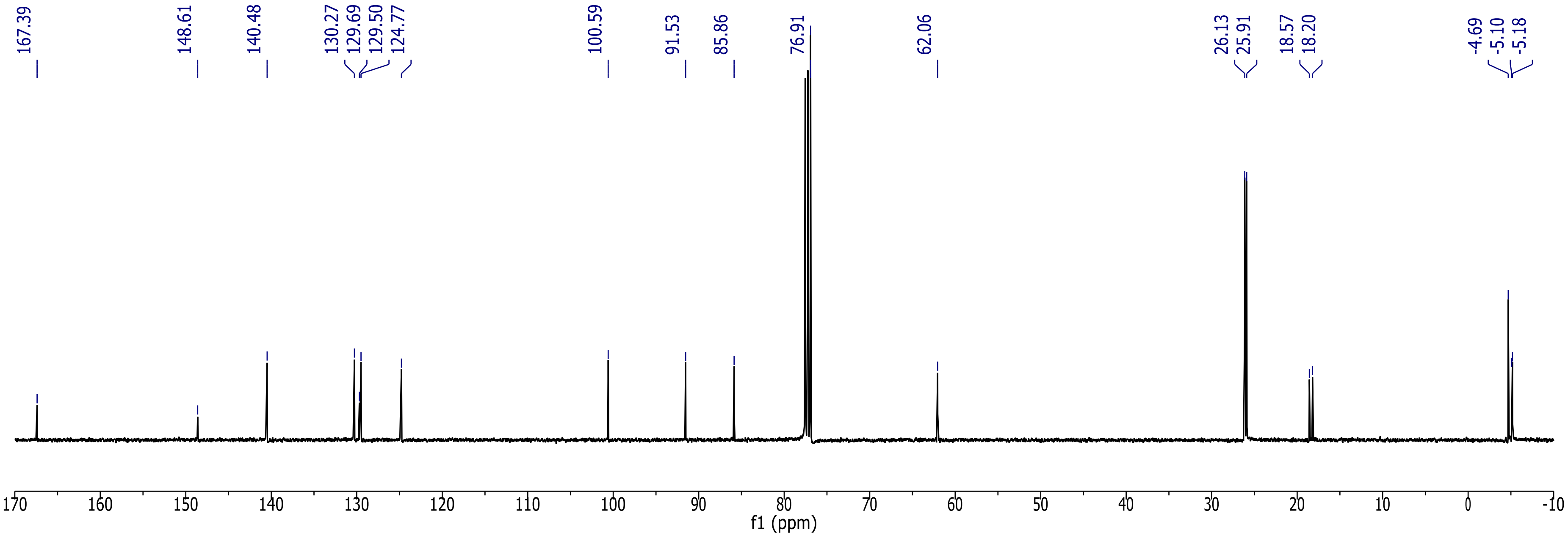
167.39
148.61
140.48
130.27
129.69
129.50
124.77
100.59
91.53
85.86
76.91
62.06
26.13
25.91
18.57
18.20
-4.69
-5.10
-5.18
170
160
150
140
130
120
110
100
90
80
70
60
50
40
30
20
10
0
-10
f1 (ppm)

Figure S23. **2-[(3-Nitrophenyl)sulfanyl]-4,5-dihydro(3′,5′-di-*O*-*tert*-1′,2′-dideoxy-*β*-*D*-arabinofuranoso)-[1,2-*d*]-oxazole (16). ^{1}H NMR (400 MHz, $CDCl_3$) and ^{13}C NMR (100 MHz, $CDCl_3$) spectrums.**

Acquisition Parameter

Source Type	ESI	Ion Polarity	Positive	Set Nebulizer	0.6 Bar
Focus	Not active	Set Capillary	4500 V	Set Dry Heater	200 °C
Scan Begin	50 m/z	Set End Plate Offset	-500 V	Set Dry Gas	7.0 l/min
Scan End	3000 m/z	Set Collision Cell RF	1000.0 Vpp	Set Divert Valve	Waste

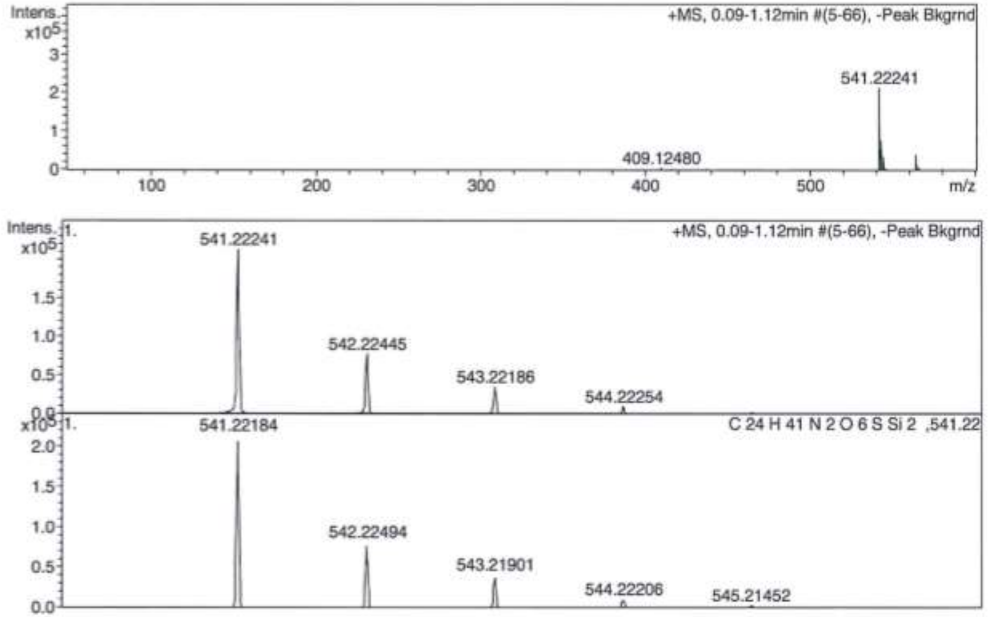

Meas. m/z	#	Formula	m/z	err [ppm]	mSigma	rdb	e⁻ Conf	N-Rule
541.22241	1	C 24 H 41 N 2 O 6 S Si 2	541.22184	-1.06	17.9	7.5	even	ok
563.20412	1	C 24 H 40 N 2 Na O 6 S Si 2	563.20378	-0.60	20.3	7.5	even	ok

Figure S24. 2-[(3-Nitrophenyl)sulfanyl]-4,5-dihydro(3′,5′-di-*O*-*tert*-1′,2′-dideoxy-*β*-*D*-arabinofuranoso)-[1,2-*d*]-oxazole (16). HRMS (ESI).

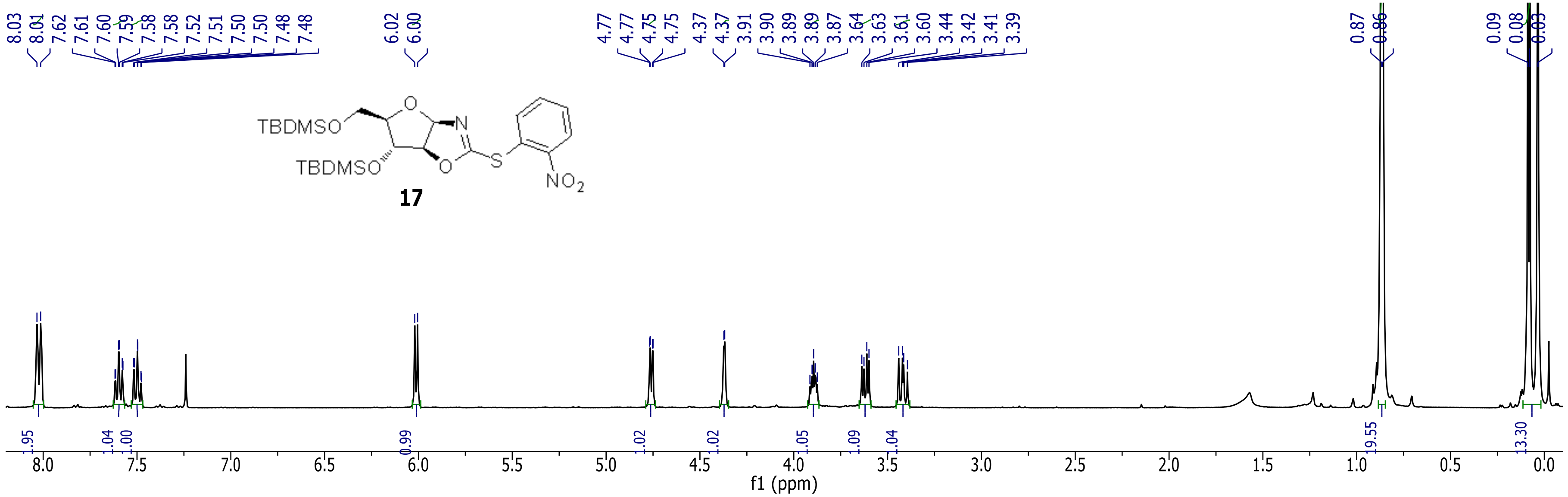
8.03 8.01 7.62 7.61 7.60 7.59 7.58 7.58 7.52 7.51 7.50 7.50 7.48 7.48
6.02 6.00
4.77 4.77 4.75 4.75 4.37 4.37 3.91 3.90 3.89 3.89 3.87 3.64 3.63 3.61 3.60 3.44 3.42 3.41 3.39
0.87 0.86
0.09 0.08 0.03
TBDMSO
TBDMSO
N
O
O
S
NO2
17
1.95 1.04 1.00 0.99 1.02 1.02 1.05 1.09 1.04 19.55 13.30
f1 (ppm)

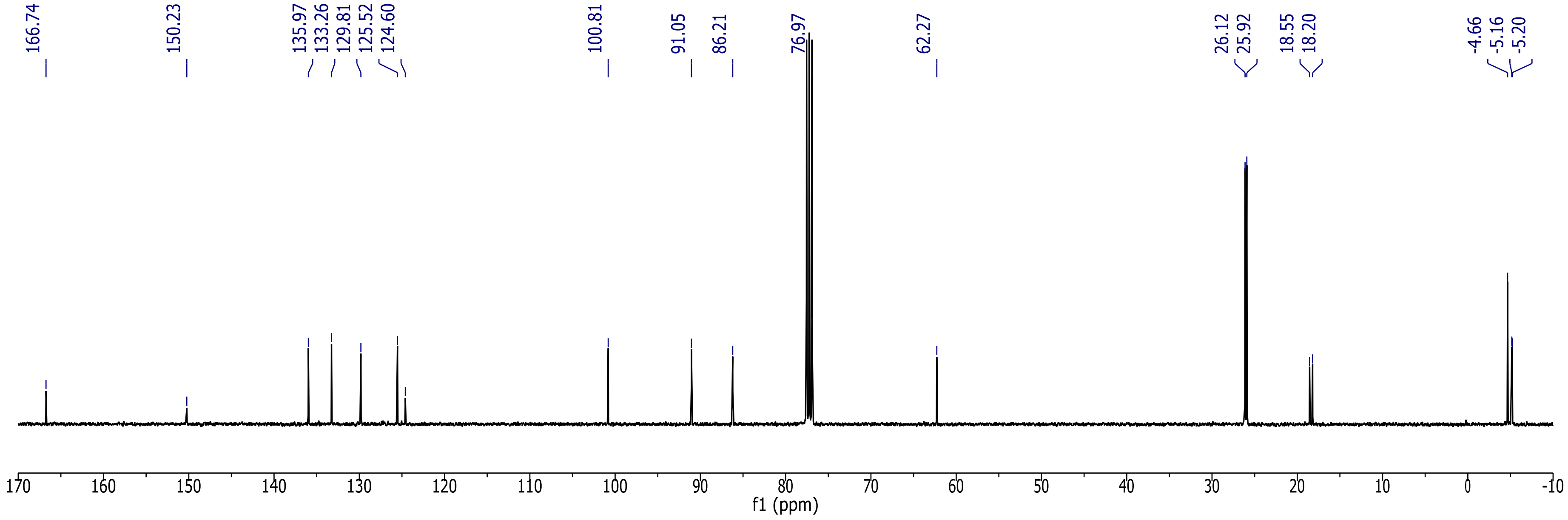
166.74
150.23
135.97 133.26 129.81 125.52 124.60
100.81
91.05
86.21
76.97
62.27
26.12 25.92
18.55 18.20
-4.66 -5.16 -5.20
f1 (ppm)

Figure S25. **2-[(2-Nitrophenyl)sulfanyl]-4,5-dihydro(3′,5′-di-*O*-*tert*-1′,2′-dideoxy-*β*-*D*-arabinofuranoso)-[1,2-*d*]-oxazole (17).** **^{1}H NMR (400 MHz, $CDCl_3$) and ^{13}C NMR (100 MHz, $CDCl_3$) spectrums.**

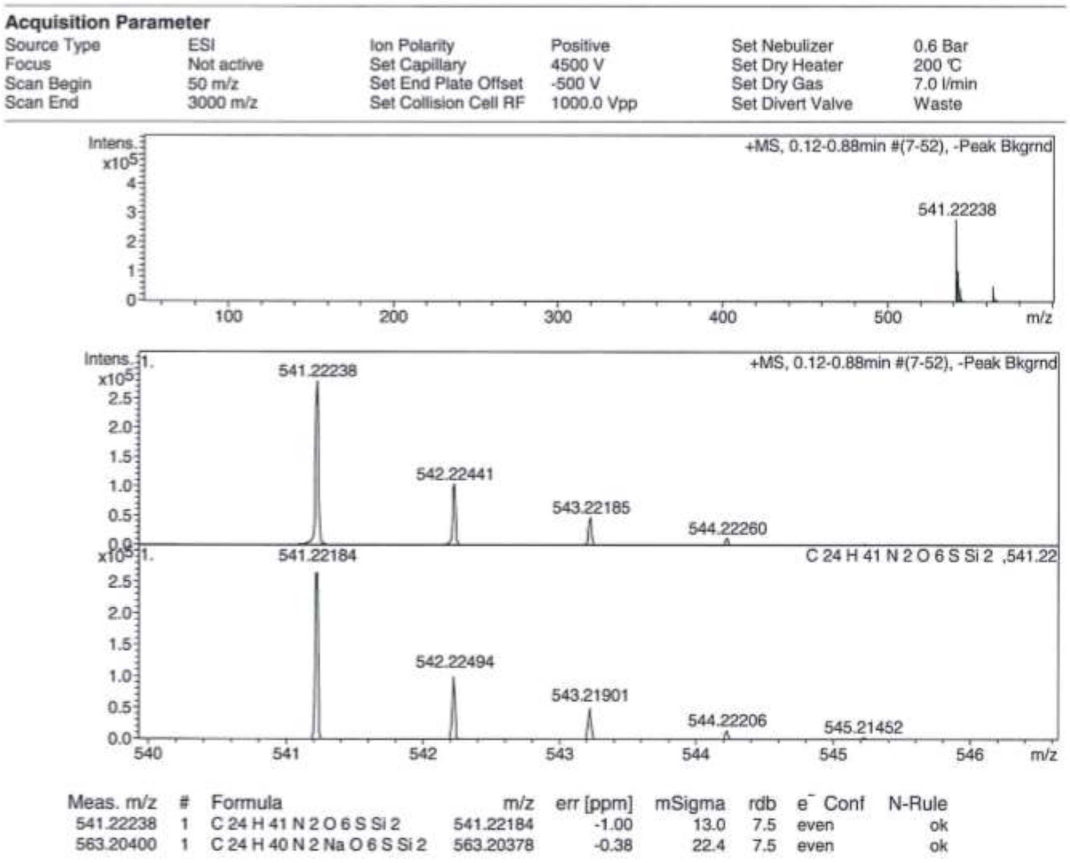

Figure S26. **2-[(2-Nitrophenyl)sulfanyl]-4,5-dihydro(3′,5′-di-*O*-*tert*-1′,2′-dideoxy-*β*-*D*-arabinofuranoso)-[1,2-*d*]-oxazole (17). HRMS (ESI).**

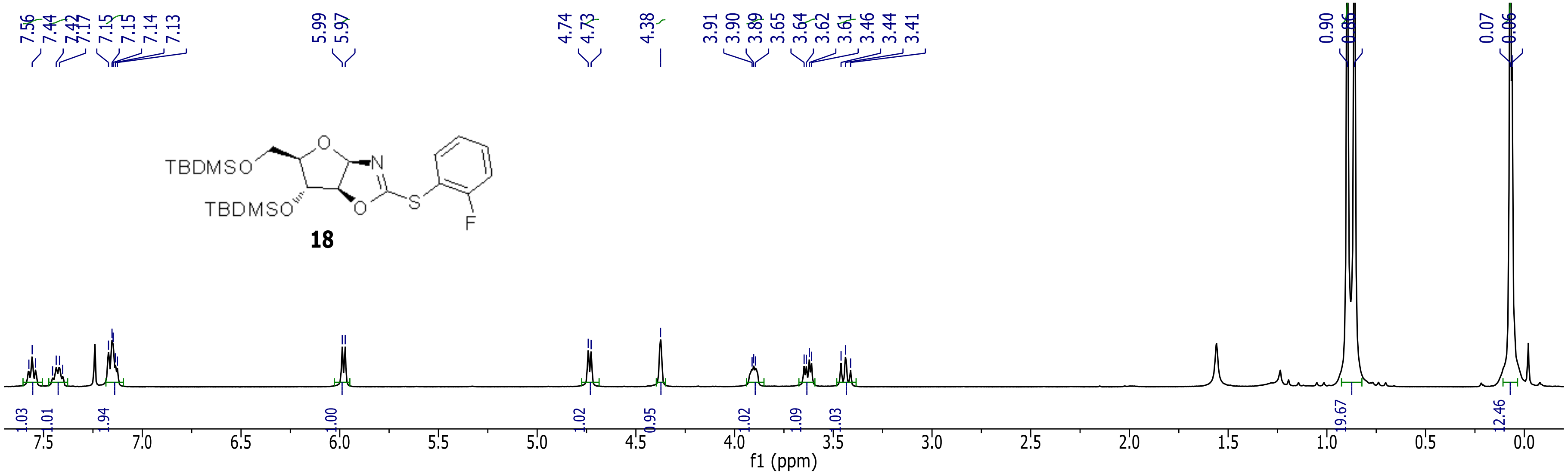

7.56
7.44
7.42
7.17
7.15
7.15
7.14
7.13
5.99
5.97
4.74
4.73
4.38
3.91
3.90
3.89
3.65
3.64
3.62
3.61
3.46
3.44
3.41
0.90
0.07
TBDMSO
TBDMSO
O
N
O
S
F
18
1.03
1.01
1.94
1.00
1.02
0.95
1.02
1.09
1.03
19.67
12.46
7.5
7.0
6.5
6.0
5.5
5.0
4.5
4.0
3.5
3.0
2.5
2.0
1.5
1.0
0.5
0.0
f1 (ppm)

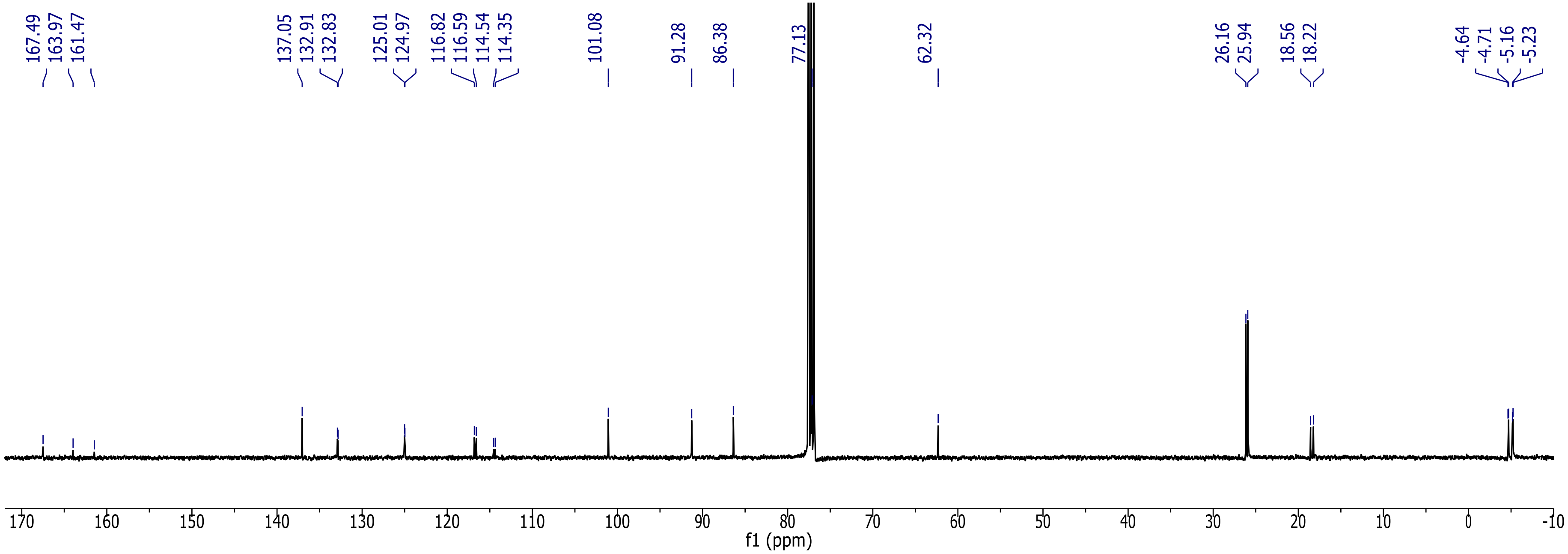

167.49
163.97
161.47
137.05
132.91
132.83
125.01
124.97
116.82
116.59
114.54
114.35
101.08
91.28
86.38
77.13
62.32
26.16
25.94
18.56
18.22
-4.64
-4.71
-5.16
-5.23
170
160
150
140
130
120
110
100
90
80
70
60
50
40
30
20
10
0
-10
f1 (ppm)

Figure S27. 2-[(2-Fluorophenyl)sulfanyl]-4,5-dihydro(3′,5′-di-*O*-*tert*-1′,2′-dideoxy-*β*-*D*-arabinofuranoso)-[1,2-*d*]-oxazole (18). ^{1}H NMR (400 MHz, $CDCl_3$) and ^{13}C NMR (100 MHz, $CDCl_3$) spectrums.

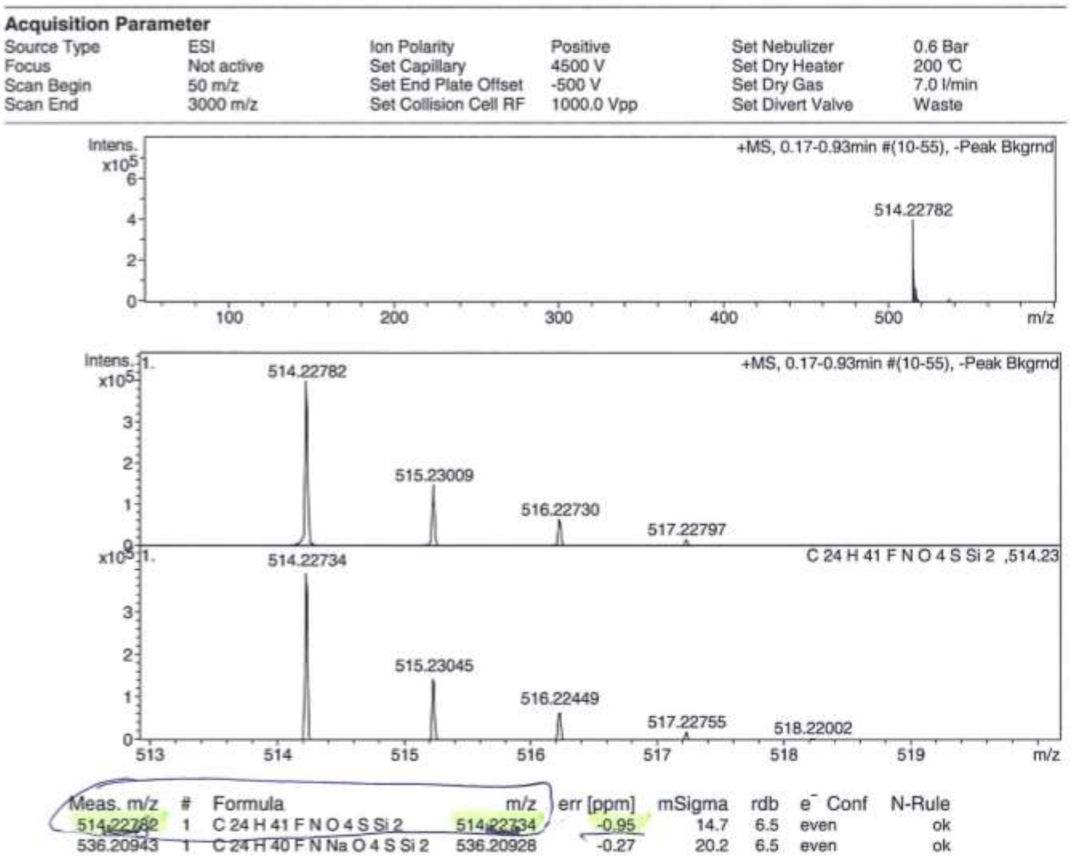

Figure S28. 2-[(2-Fluorophenyl)sulfanyl]-4,5-dihydro(3′,5′-di-*O*-*tert*-1′,2′-dideoxy-*β*-*D*-arabinofuranoso)-[1,2-*d*]-oxazole (18). HRMS (ESI).

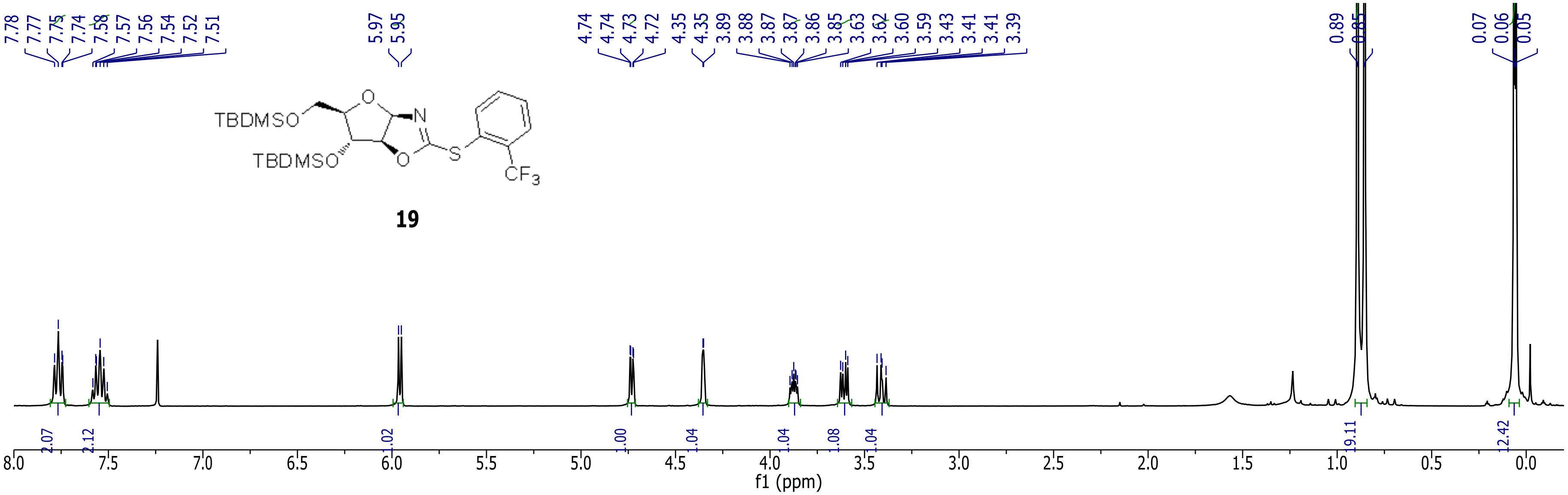

7.78 7.77 7.75 7.74 7.58 7.57 7.56 7.54 7.52 7.51
5.97 5.95
4.74 4.74 4.73 4.72 4.35 4.35 3.89 3.88 3.87 3.87 3.86 3.85 3.63 3.62 3.60 3.59 3.43 3.41 3.41 3.39
0.89 0.85
0.07 0.06 0.05
TBDMSO
TBDMSO
O
N
O
S
CF3
2.07 2.12 1.02 1.00 1.04 1.04 1.08 1.04 19.11 12.42
8.0 7.5 7.0 6.5 6.0 5.5 5.0 4.5 4.0 3.5 3.0 2.5 2.0 1.5 1.0 0.5 0.0
f1 (ppm)

19

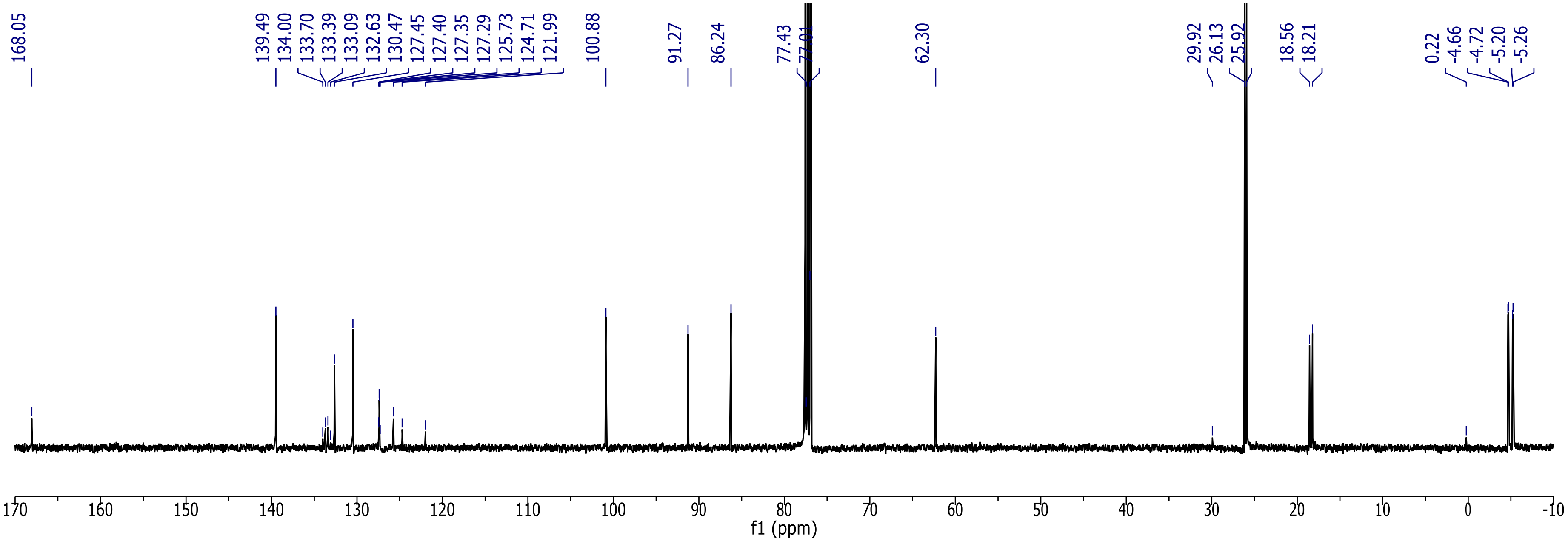

168.05
139.49 134.00 133.70 133.39 133.09 132.63 130.47 127.45 127.40 127.35 127.29 125.73 124.71 121.99
100.88
91.27
86.24
77.43 77.01
62.30
29.92 26.13 25.92
18.56 18.21
0.22 -4.66 -4.72 -5.20 -5.26
170 160 150 140 130 120 110 100 90 80 70 60 50 40 30 20 10 0 -10
f1 (ppm)

Figure S29. 2-[(2-Trifluoromethylphenyl)sulfanyl]-4,5-dihydro(3′,5′-di-*O*-*tert*-1′,2′-dideoxy-*β*-*D*-arabinofuranoso)-[1,2-*d*]-oxazole (19). ^{1}H NMR (400 MHz, $CDCl_3$) and ^{13}C NMR (100 MHz, $CDCl_3$) spectrums.

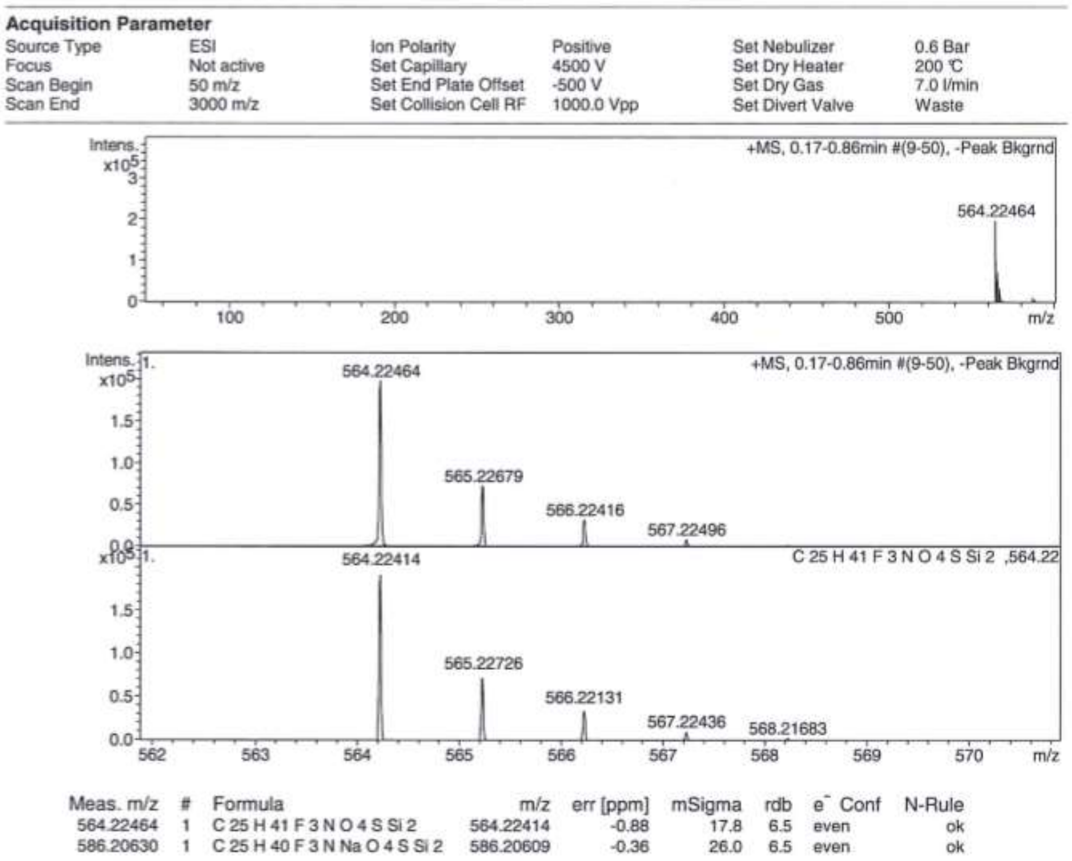

Figure S30. 2-[(2-Trifluoromethylphenyl)sulfanyl]-4,5-dihydro(3′,5′-di-*O*-*tert*-1′,2′-dideoxy-*β*-*D*-arabinofuranoso)-[1,2-*d*]-oxazole (19). HRMS (ESI).

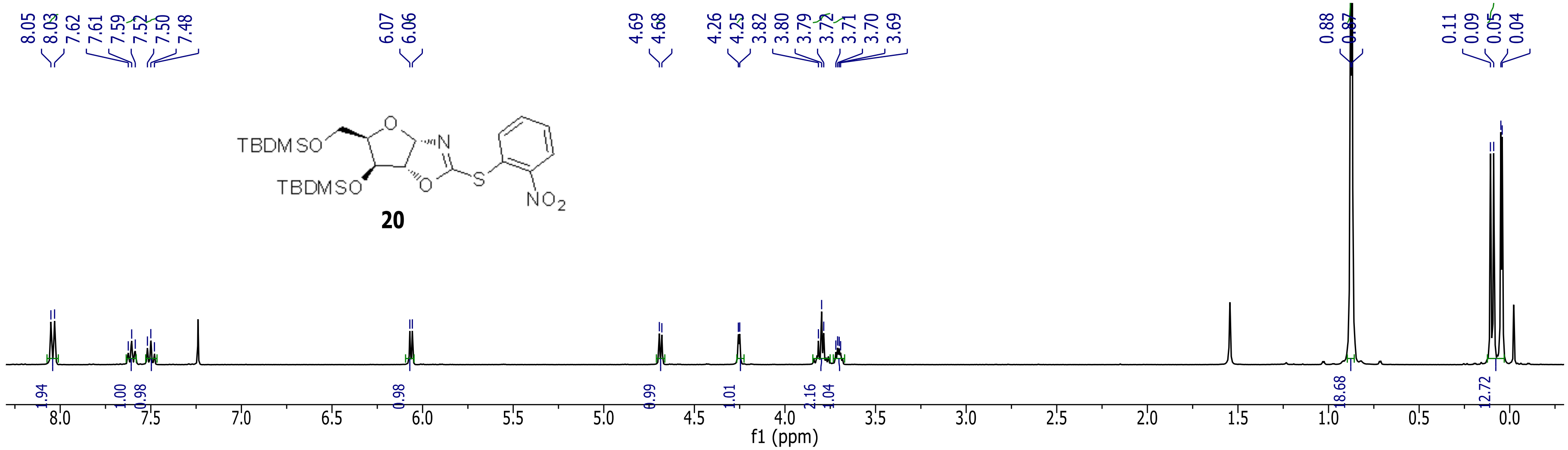
8.05
8.03
7.62
7.61
7.59
7.52
7.50
7.48
6.07
6.06
4.69
4.68
4.26
4.25
3.82
3.80
3.79
3.72
3.71
3.70
3.69
0.88
0.87
0.11
0.09
0.05
0.04
TBDMSO
TBDMSO
N
O
S
NO_2
20
1.94
1.00
0.98
0.98
0.99
1.01
2.16
1.04
18.68
12.72
f1 (ppm)

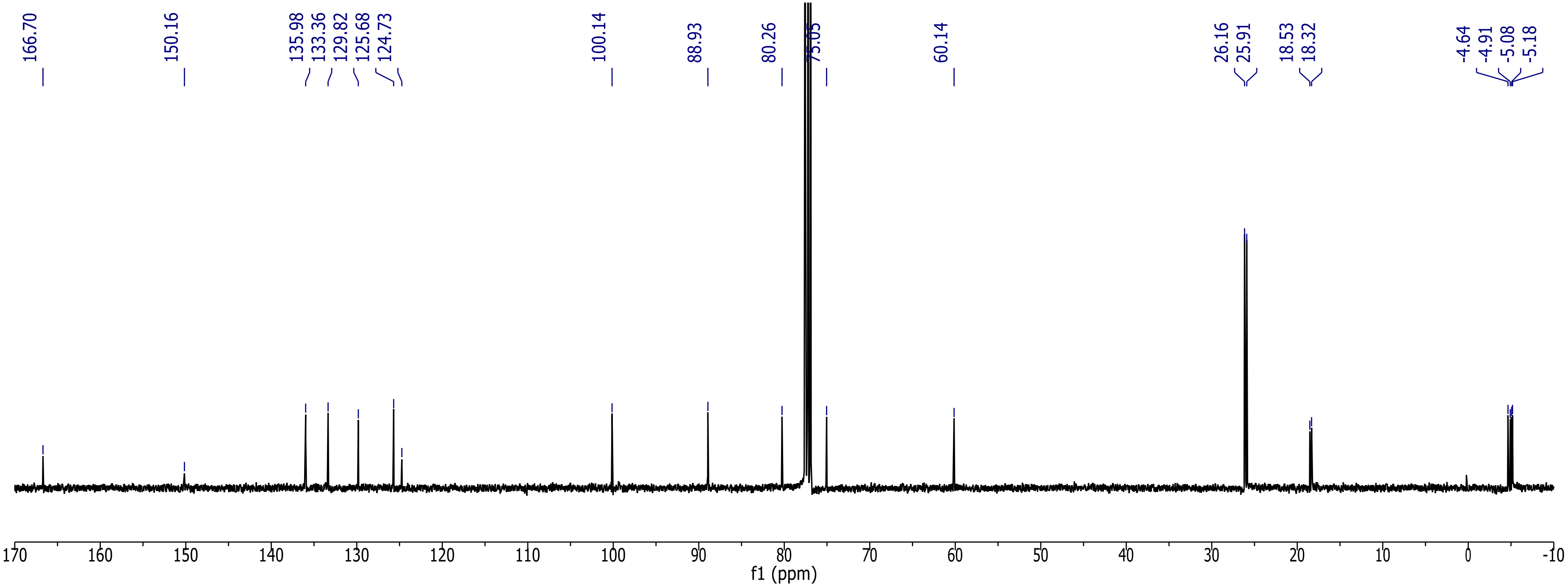
166.70
150.16
135.98
133.36
129.82
125.68
124.73
100.14
88.93
80.26
75.05
60.14
26.16
25.91
18.53
18.32
-4.64
-4.91
-5.08
-5.18
f1 (ppm)

Figure S31. **2-[(2-Nitrophenyl)sulfanyl]-4,5-dihydro(3′,5′-di-*O*-*tert*-1′,2′-dideoxy-*β*-*D*- xylofuranoso)-[1,2-*d*]-oxazole (20).** **^{1}H NMR (400 MHz, $CDCl_3$) and ^{13}C NMR (100 MHz, $CDCl_3$) spectrums.**

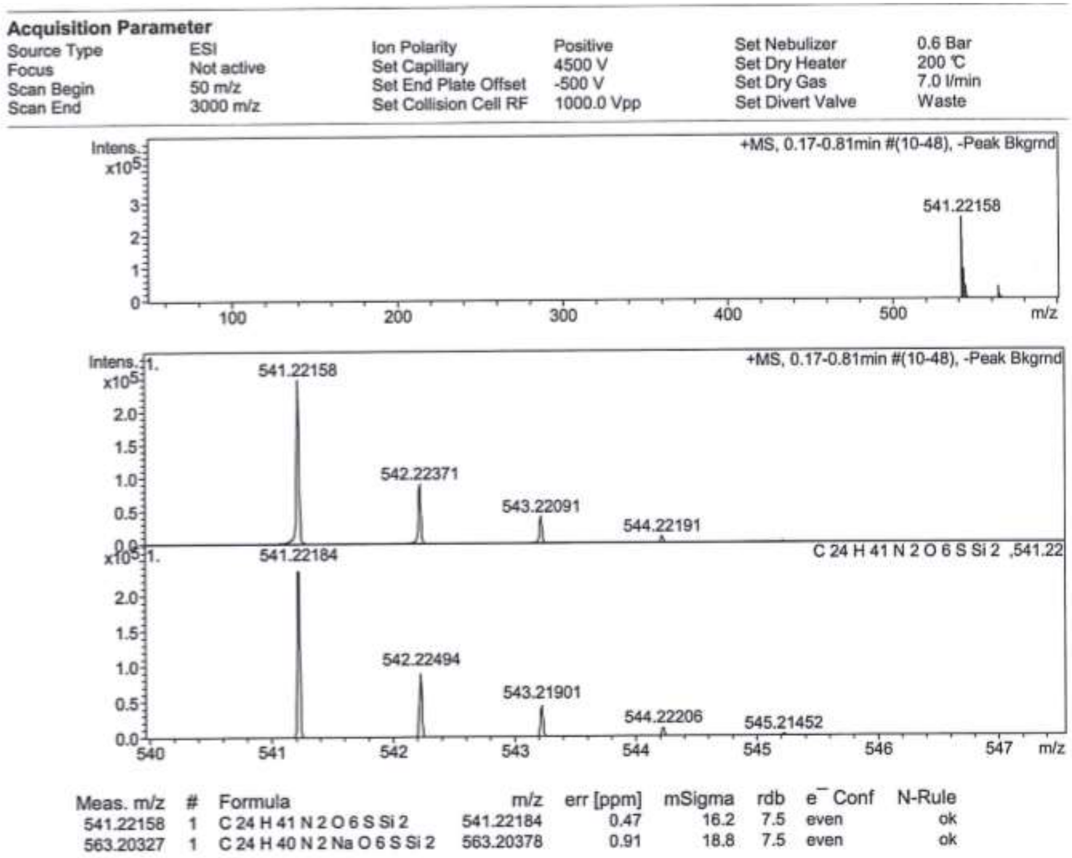

Figure S32. **2-[(2-Nitrophenyl)sulfanyl]-4,5-dihydro(3′,5′-di-*O*-*tert*-1′,2′-dideoxy-*β*-*D*- xylofuranoso)-[1,2-*d*]-oxazole (20). HRMS (ESI).**

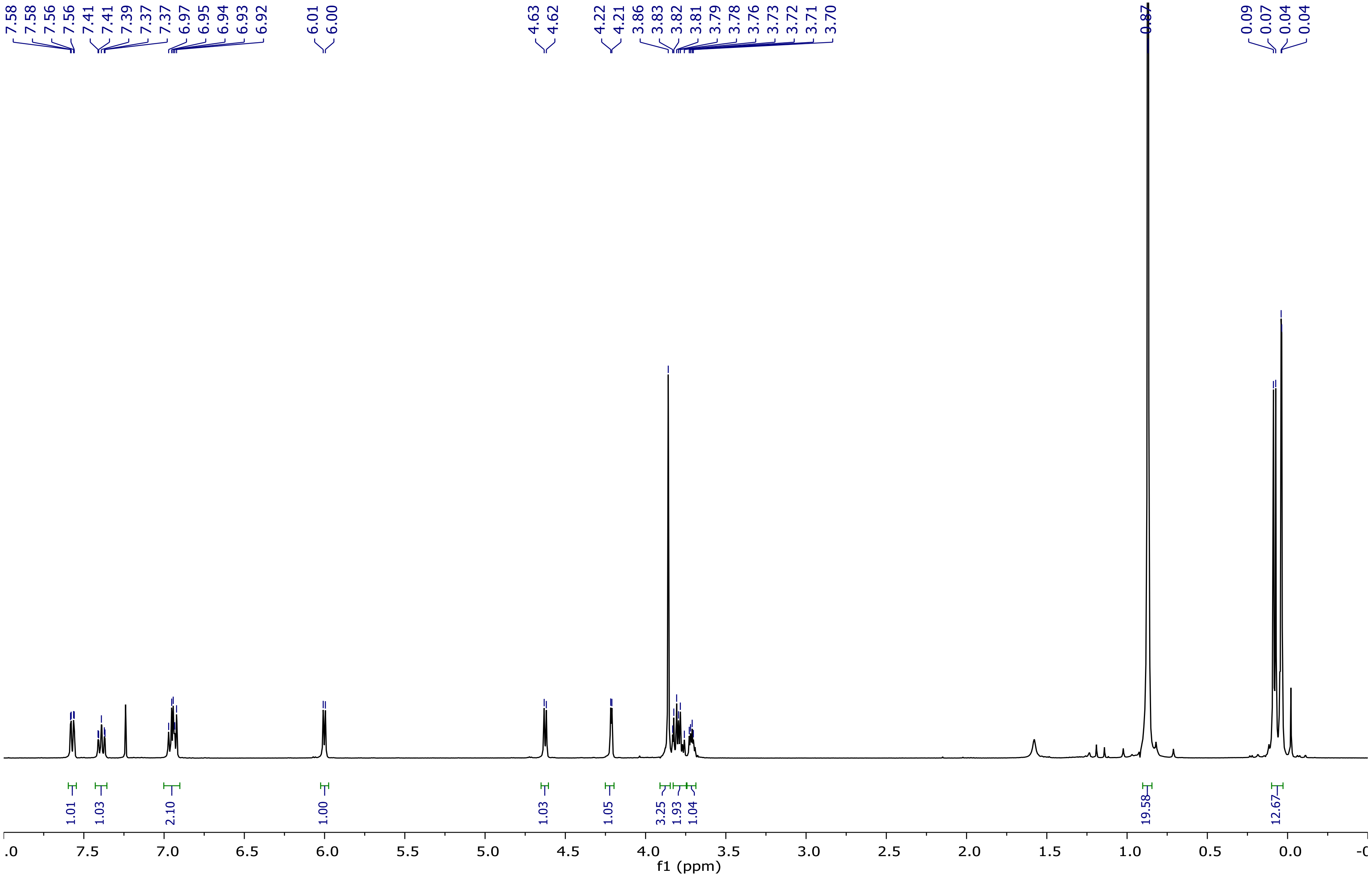

Figure S33. 2-[(2-Methoxyphenyl)sulfanyl]-4,5-dihydro(3′,5′-di-*O-tert*-1′,2′-dideoxy-*β-D*- xylofuranoso)-[1,2-*d*]-oxazole (21). ^{1}**H NMR (400 MHz,** $CDCl_3$**) spectrum.**

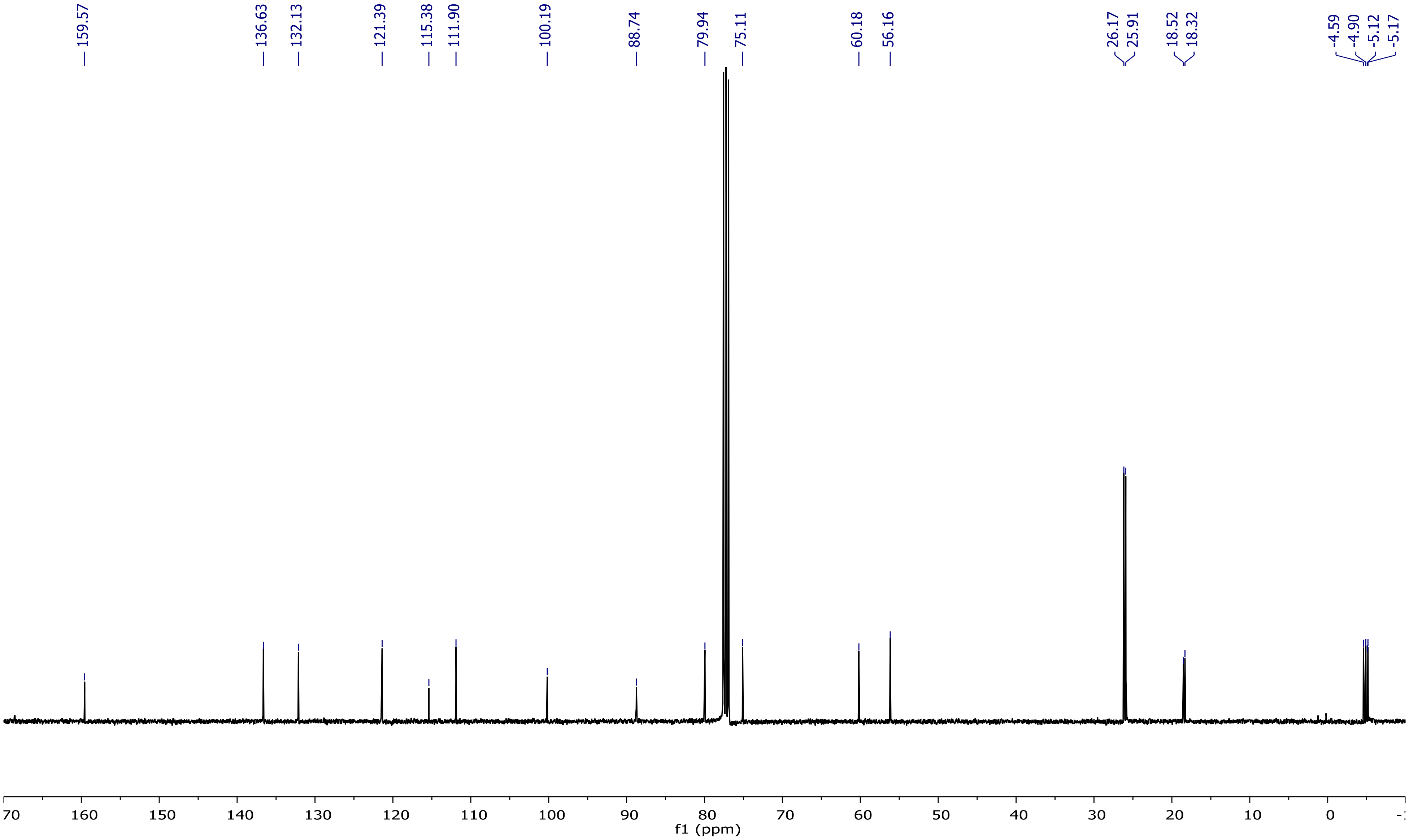

Figure S34. 2-[(2-Methoxyphenyl)sulfanyl]-4,5-dihydro(3′,5′-di-*O*-*tert*-1′,2′-dideoxy-*β*-*D*- xylofuranoso)-[1,2-*d*]-oxazole (21). ^{13}C NMR (100 MHz, $CDCl_3$) spectrum.

Acquisition Parameter

Source Type	ESI	Ion Polarity	Positive	Set Nebulizer	0.6 Bar
Focus	Not active	Set Capillary	4500 V	Set Dry Heater	200 ℃
Scan Begin	50 m/z	Set End Plate Offset	-500 V	Set Dry Gas	7.0 l/min
Scan End	3000 m/z	Set Collision Cell RF	1000.0 Vpp	Set Divert Valve	Waste

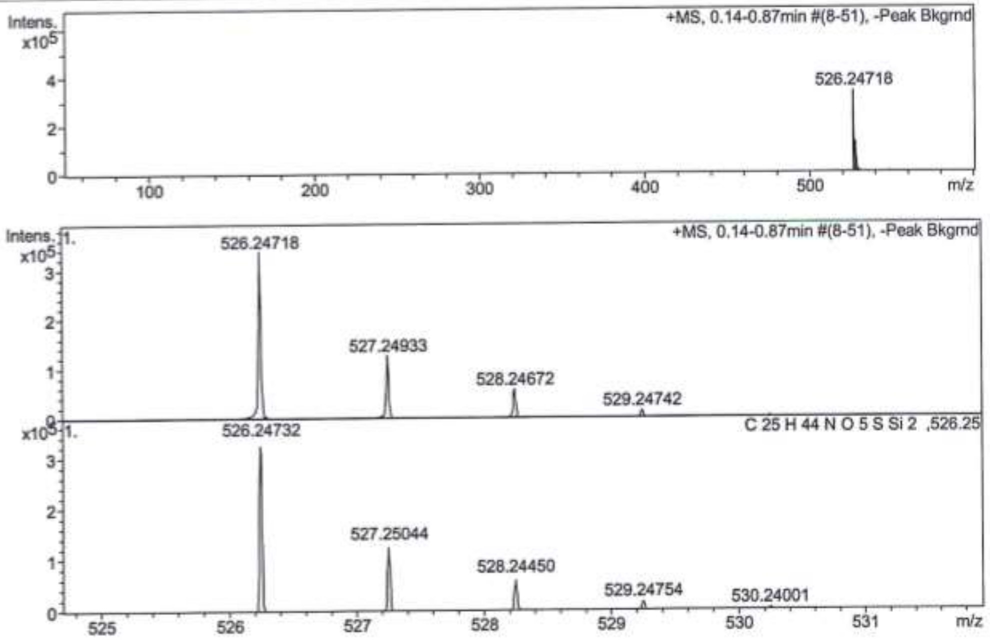

Meas. m/z	#	Formula	m/z	err [ppm]	mSigma	rdb	e⁻ Conf	N-Rule
526.24718	1	C 25 H 44 N O 5 S Si 2	526.24732	0.27	13.0	6.5	even	ok

Figure S35. 2-[(2-Methoxyphenyl)sulfanyl]-4,5-dihydro(3′,5′-di-*O*-*tert*-1′,2′-dideoxy-*β*-*D*- xylofuranoso)-[1,2-*d*]-oxazole (21). HRMS (ESI).

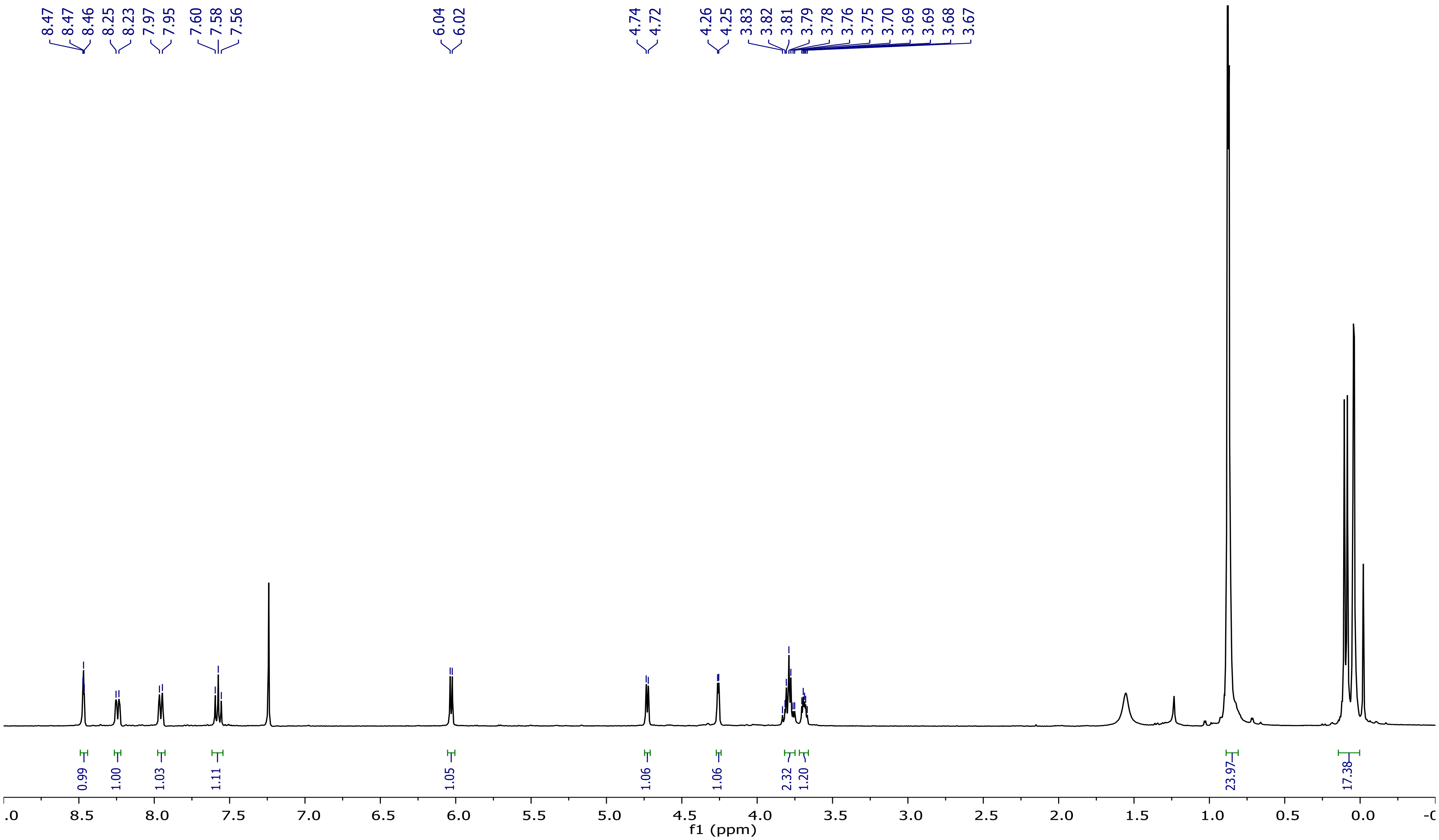

Figure S36. 2-[(2-Nitrophenyl)sulfanyl]-4,5-dihydro(3′,5′-di-*O*-*tert*-1′,2′-dideoxy-*β*-*D*-ribofuranoso)-[1,2-*d*]-oxazole (22). ^{1}H NMR (400 MHz, $CDCl_3$) spectrum.

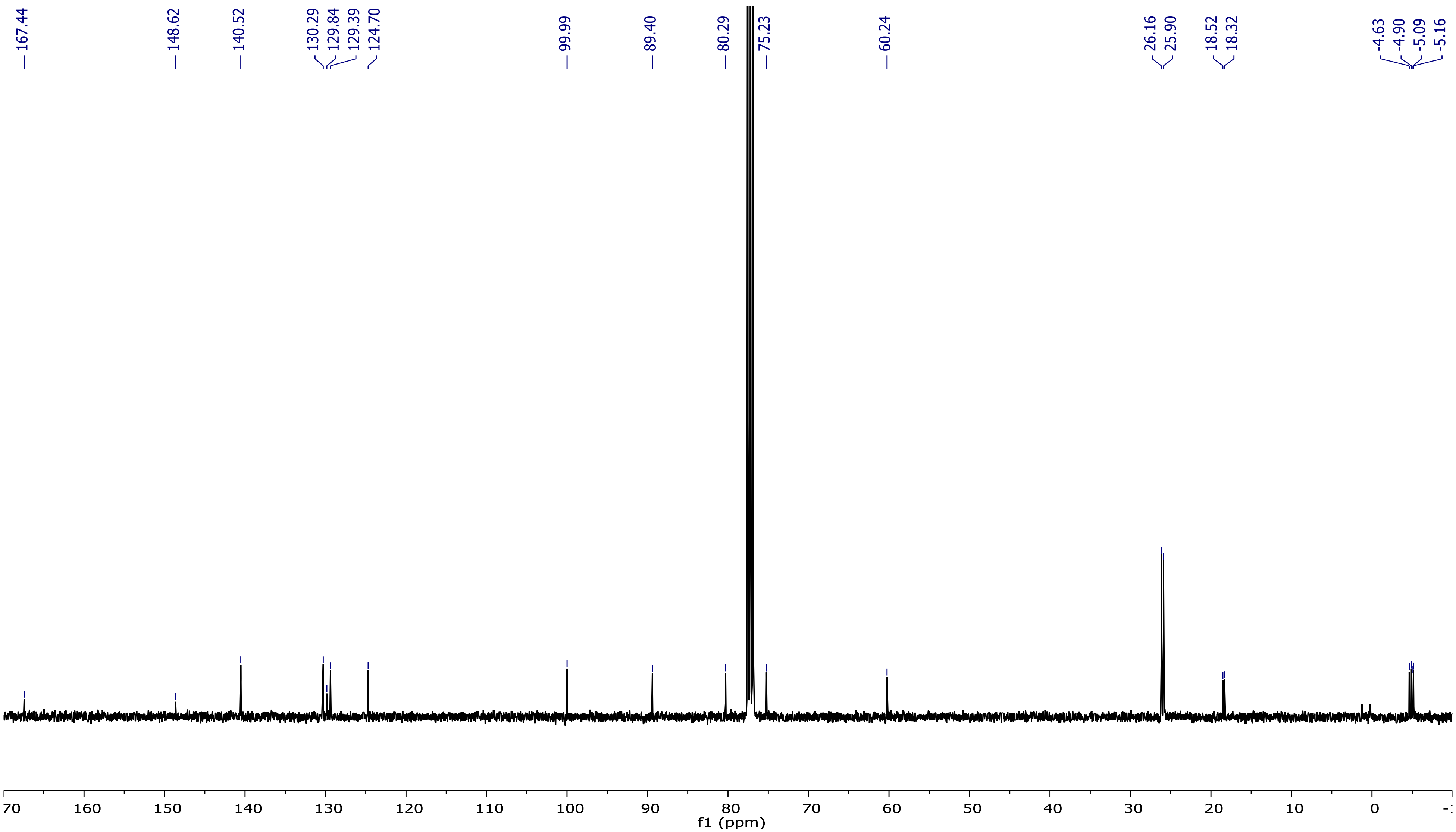

Figure S37. 2-[(2-Nitrophenyl)sulfanyl]-4,5-dihydro(3′,5′-di-*O*-*tert*-1′,2′-dideoxy-*β*-*D*-ribofuranoso)-[1,2-*d*]-oxazole (22). ^{13}C NMR (100 MHz, $CDCl_3$) spectrum.

Acquisition Parameter

Source Type	ESI	Ion Polarity	Positive	Set Nebulizer	0.6 Bar
Focus	Not active	Set Capillary	4500 V	Set Dry Heater	200 °C
Scan Begin	50 m/z	Set End Plate Offset	-500 V	Set Dry Gas	7.0 l/min
Scan End	3000 m/z	Set Collision Cell RF	1000.0 Vpp	Set Divert Valve	Waste

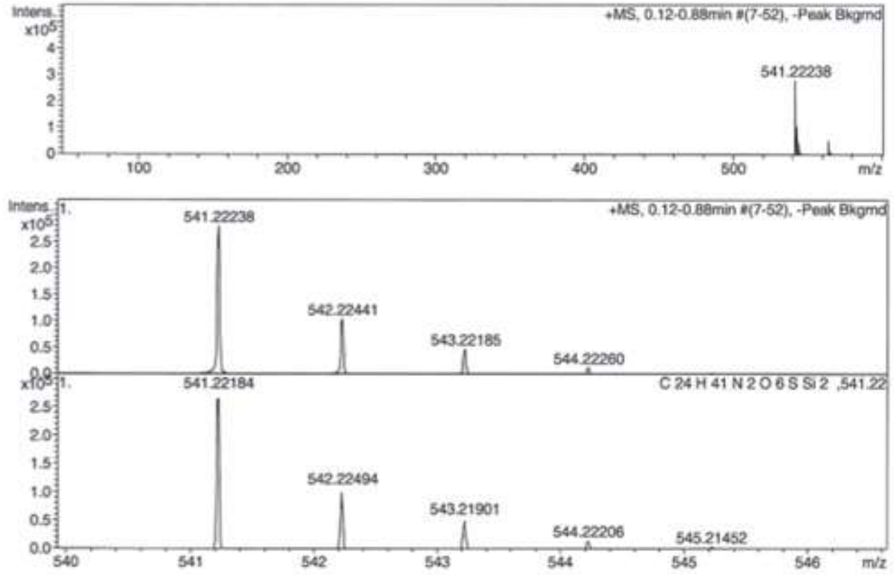

Meas. m/z	#	Formula	m/z	err [ppm]	mSigma	rdb	e⁻ Conf	N-Rule
541.22238	1	C 24 H 41 N 2 O 6 S Si 2	541.22184	-1.00	13.0	7.5	even	ok

Figure S38. 2-[(2-Nitrophenyl)sulfanyl]-4,5-dihydro(3′,5′-di-*O*-*tert*-1′,2′-dideoxy-*β*-*D*-ribofuranoso)-[1,2-*d*]-oxazole (22). HRMS (ESI).
